# Chromosome-scale assembly of the *Dendrobium chrysotoxum* genome enhances the understanding of orchid evolution

**DOI:** 10.1038/s41438-021-00621-z

**Published:** 2021-09-01

**Authors:** Yongxia Zhang, Guo-Qiang Zhang, Diyang Zhang, Xue-Die Liu, Xin-Yu Xu, Wei-Hong Sun, Xia Yu, Xiaoen Zhu, Zhi-Wen Wang, Xiang Zhao, Wen-Ying Zhong, Hongfeng Chen, Wei-Lun Yin, Tengbo Huang, Shan-Ce Niu, Zhong-Jian Liu

**Affiliations:** 1 Guangdong Provincial Key Laboratory for Plant Epigenetics, College of Life Sciences and Oceanography, Shenzhen University, Shenzhen, 518071 China; 2 Laboratory for Orchid Conservation and Utilization, Orchid Conservation and Research Center, The National Orchid Conservation Center, Shenzhen, 518114 China; 3 School of Food Science and Technology, Foshan University, Foshan, 528225 China; 4 Key Laboratory of National Forestry and Grassland Administration for Orchid Conservation and Utilization at College of Landscape Architecture, Fujian Agriculture and Forestry University, Fuzhou, 350002 China; 5 College of Forestry, Fujian Agriculture and Forestry University, Fuzhou, 350002 China; 6 PubBio-Tech, Wuhan, 430070 China; 7 Key Laboratory of Plant Resources Conservation Sustainable Utilization, South China Botanical Garden, Chinese Academy of Sciences, Guangzhou, 510650 China; 8 College of Biological Sciences and Technology, Beijing Forestry University, Beijing, 100083 China; 9 College of Horticulture, Hebei Agricultural University, Baoding, 071000 China

**Keywords:** Genome, Plant molecular biology, Gene regulation

## Abstract

As one of the largest families of angiosperms, the Orchidaceae family is diverse. *Dendrobium* represents the second largest genus of the Orchidaceae. However, an assembled high-quality genome of species in this genus is lacking. Here, we report a chromosome-scale reference genome of *Dendrobium chrysotoxum*, an important ornamental and medicinal orchid species. The assembled genome size of *D. chrysotoxum* was 1.37 Gb, with a contig N50 value of 1.54 Mb. Of the sequences, 95.75% were anchored to 19 pseudochromosomes. There were 30,044 genes predicted in the *D. chrysotoxum* genome. Two whole-genome polyploidization events occurred in *D. chrysotoxum*. In terms of the second event, whole-genome duplication (WGD) was also found to have occurred in other Orchidaceae members, which diverged mainly via gene loss immediately after the WGD event occurred; the first duplication was found to have occurred in most monocots (tau event). We identified sugar transporter (*SWEET*) gene family expansion, which might be related to the abundant medicinal compounds and fleshy stems of *D. chrysotoxum*. MADS-box genes were identified in *D. chrysotoxum*, as well as members of TPS and Hsp90 gene families, which are associated with resistance, which may contribute to the adaptive evolution of orchids. We also investigated the interplay among carotenoid, ABA, and ethylene biosynthesis in *D. chrysotoxum* to elucidate the regulatory mechanisms of the short flowering period of orchids with yellow flowers. The reference *D. chrysotoxum* genome will provide important insights for further research on medicinal active ingredients and breeding and enhances the understanding of orchid evolution.

## Introduction

With more than 25,000 species, Orchidaceae is the largest angiosperm family^1^ and comprises 8–10% of flowering plants. Orchids are renowned for their specialized flowers, which have a very wide variety of growth forms, and have been successful colonizers of a wide variety of different habitats^2^. As one of the largest genera of Orchidaceae, *Dendrobium* encompasses ~1450 species with fleshy stems^3^. Many species of *Dendrobium* have high medicinal and commercial value, and the main medicinal active ingredients are in the stems^4–9^. Therefore, studying the molecular mechanism of these active ingredients and breeding cultivars with increased contents of natural products are the main objectives in *Dendrobium* scientific research and industrialization^10^.

**Fig. 1:**
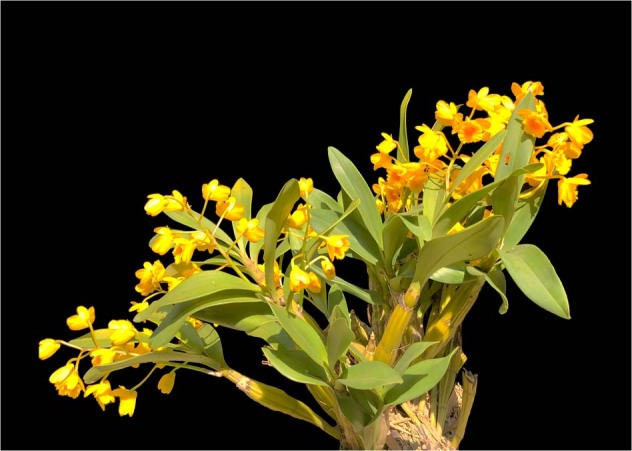
A flowering *D. chrysotoxum* plant. *D. chrysotoxum* is lithophytic on rocks or epiphytic on tree trunks with bright yellow flowers and fusiform, fleshy stems

Guchui Shihu (鼓槌石斛) *Dendrobium chrysotoxum*, a medicinal species, is listed in the Chinese Pharmacopoeia (2020, 2015, and 2010 edition) and contains an abundance of erianin, gigantol, polysaccharides, and fluorenones, among other compounds^11–19^ (Fig. 1). These compounds show antipyretic, analgesic, antihyperglycemic, and antioxidant effects and enhance immune function^11–19^. Recently, preliminary clinical study results suggested that gigantol could delay lens turbidity through the inhibition of aldose reductase and aldose reductase mRNA expression, which have good effects on diabetic cataracts^16,17^. Erianin has been demonstrated to exhibit metabolic inhibition^20^ and antitumor^21^, antiproliferative^22^, and antiangiogenic activity^23^. Moreover, it also inhibits high glucose-induced retinal angiogenesis^12^. Polysaccharides isolated from *D. chrysotoxum* have potential utility in enhancing antioxidation, immune function, and/or hypoglycemic activity^11^. The stems of *D. chrysotoxum* are fusiform and rich in active medicinal substances, which makes it a suitable species for scientific research and industrial applications in *Dendrobium*.

With the improvement of sequencing technology and cost reduction, genome sequencing has become a necessary method for obtaining comprehensive genetic information and an effective method for screening candidate genes for specific traits, especially for identifying candidate genes involved in the biosynthesis pathways of medicinal compounds^24–28^. To date, only two *Dendrobium* spp. genomes have been sequenced, and some candidate genes involved in polysaccharide metabolic pathways have been identified in those two species^24,29,30^. However, these studies were largely limited due to their low-quality genome assemblies. Therefore, high-quality reference genomes and additional *Dendrobium* species need to be sequenced to better understand the molecular mechanisms underlying the production of medicinal compounds and enable the breeding of new varieties.

In this study, we used PacBio sequencing and Hi-C technology to generate a chromosome-level genome assembly. The specific genes of *D. chrysotoxum* were identified, which lays a foundation for further research on the functions of medicinal active ingredients, provides a reference for breeding new varieties and enhances the understanding of orchid evolution.

## Results and discussion

### Genome sequencing and characteristics

**Fig. 2:**
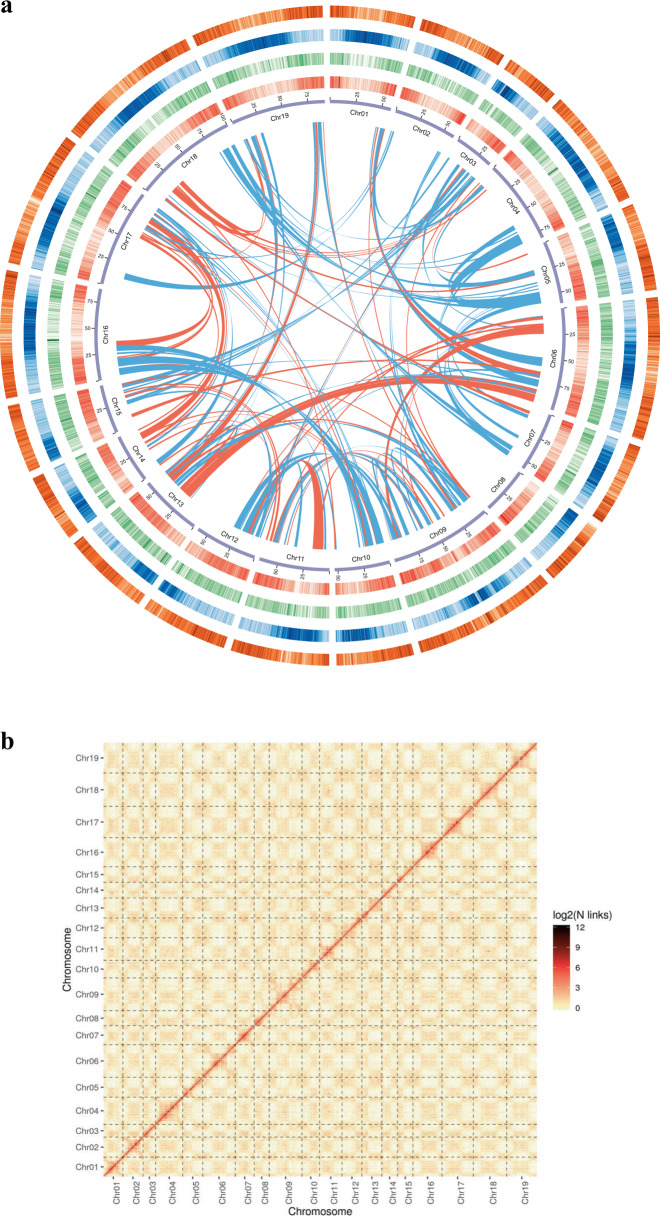
Chromosomal features and intensity signal heat map of *D. chrysotoxum* chromosomes according to Hi-C output. **a** From inside outward: chromosome (purple), gene density (red), DNA type repeat sequence density (green), copy density (blue), and gypsy density (orange). All the data are shown with sliding windows of 500 kb, and the inner lines (green indicates the positive direction, and red indicates the opposite direction) represent syntenic blocks on homologous chromosomes. **b** Heat map of the intensity of the Hi-C chromosome. The heat map represents the contact matrices generated by aligning the Hi-C data to the chromosome-scale assembly of the *D. chrysotoxum* genome. A higher value on the scale bar indicates a higher contact frequency

*D. chrysotoxum* has a karyotype of 2N = 2X = 38, with uniform chromosomes^31^. To completely sequence the *D. chrysotoxum* genome, 138.15 Gb of clean reads were generated by BGISEQ sequencing system (Supplementary Table 1). The estimated genome size was 1.38 Gb with 1.84% heterozygosity, as determined by K-mer analysis (Supplementary Fig. 1). To obtain a better assembly, PacBio technology was employed, and 132.64 Gb of PacBio sequencing data were generated (Supplementary Table 1). The assembly size was 1.37 Gb with a corresponding contig N50 value of 1.54 Mb (Supplementary Table 2). The BUSCO^32^ assessment indicated that the completeness of the gene set of the assembled genome was 90.3% (Supplementary Table 3). This indicates that the *D. chrysotoxum* genome assembly was complete and could be used for subsequent analysis. We further used 125.96 Gb of reads from the Hi-C library. The assembled scaffolds were ultimately clustered into 19 pseudomolecules, which represented the 19 chromosomes in the haploid genome of *D. chrysotoxum* (Fig. 2a). The lengths of the 19 pseudochromosomes ranged from 38.28 to 100.49 Mb with a scaffold N50 value of 67.80 Mb (Supplementary Tables 4 and 5). In addition, contigs with a length of 1.31 Gb were mapped onto the 19 pseudochromosomes at a 95.75% anchor rate (Supplementary Tables 4 and 5). The chromatin interaction data suggest that our Hi-C assembly is of high quality (Fig. 2b). Compared with those of other orchid genome assemblies, the contig N50 and scaffold N50 values of the *D. chrysotoxum* genome were much higher (Table 1), and the assembly completeness was higher than 90% (Table 1), suggesting high genome quality and completeness.

**Table 1:** Genome statistics and comparisons among orchid species whose genome has been sequenced

Species	Gene number	Contig N50 (bp)	Scaffold N50 (bp)	BUSCO assembly (%)	CEGMA assembly (%)
*D. chrysotoxum*^a^	30044	1,540,953	67,798,029	90.30	**–**
*D. catenatum*^2^	28910	51,736	1,055,340	92.46	–
*P. equestris*^33^	29431	45,791	1,217,477	91.00	–
*A. shenzhenica*^2^	21841	80,069	3,029,156	93.62	–
*D. officinale*^29^	35567	25,122	76,489	-	91.50

^a^This study

### Gene prediction and annotation

In *D. chrysotoxum* genome, 30,044 protein-coding genes were annotated (see Materials and methods; Supplementary Table 6). The completeness of the genome was 95.64%, indicating that the *D. chrysotoxum* genome annotation was relatively complete (Supplementary Table 7).

In addition to a high number of genes, the average length of genes and introns was also larger in *D. chrysotoxum* than in *Phalaenopsis equestris*, *Gastrodia elata*, and *D. catenatum*^24,33,34^ and much higher than that in most other angiosperms (Supplementary Table 8). The average length of the coding DNA sequences (CDSs) in *D. chrysotoxum* was longer than those in other angiosperms, and a greater average intron length was also previously observed for *P. equestris*, *G. elata*, and *D. catenatum*^24,33,34^; thus, a relatively long CDS might be a unique characteristic of Orchidaceae (Supplementary Fig. 2; Supplementary Table 8). Regulatory elements are frequently present in introns, and alternative splicing events often occur among different introns and exons, diversifying the protein-coding aspect of the genome. All these factors might contribute to genome structure evolution, genome size, gene function diversification, and gene expression patterns^35–38^. For example, intron transcriptional delay in *Drosophila* is particularly important for proper development of the embryo^39,40^. Thus, this characteristic of orchids needs to be further analyzed and researched. Moreover, 80 microRNAs, 1281 transfer RNAs, 2275 ribosomal RNAs, and 882 small nuclear RNAs were identified in the *D. chrysotoxum* genome (Supplementary Table 9).

We estimated that the *D. chrysotoxum* genome comprised 62.81% repetitive sequences (Supplementary Figs. 3 and 4; Supplementary Table 10), the percentage of which was higher than 62% in *P. equestris* but lower than 78.1% in *D. catenatum*^24,33^. Transposable elements (TEs) are important forms of repeats and constitute a substantial part of the *D. chrysotoxum* genome (61.22%); TEs are the most abundant repeat subtypes in this species. In addition, repeats predicted de novo were much larger than those obtained based on Repbase11 database, suggesting that, compared with other plants species whose genome has been sequenced, *D. chrysotoxum* has many specific repeats (Supplementary Table 10). Long terminal repeats (LTRs) represented the highest proportion among all subtypes of repeats, accounting for ~53.15% of the genome, which was higher than the 46% for *D. catenatum*^24^ (Supplementary Table 11).

In addition, 27,575 (91.78%) predicted genes were functionally annotated (Supplementary Table 12). Among them, 27,268 (90.76%) and 26,808 (89.23%) genes were annotated to the TrEMBL and Nr databases, respectively (Supplementary Table 12). The numbers of annotated genes were 22,735 (75.67%), 19,185 (63.86%), and 18,666 (62.13%) in the InterPro, SwissProt, and KEGG databases, respectively (Supplementary Table 12).

### Evolution of gene families

**Fig. 3:**
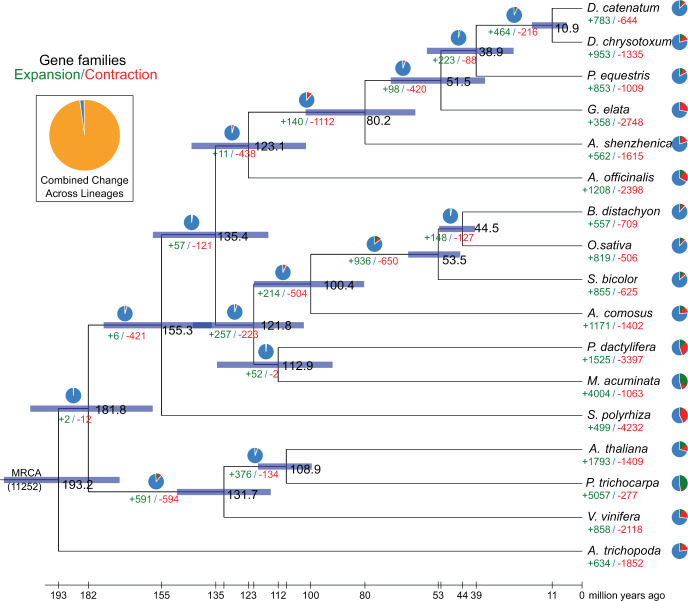
Phylogenetic tree showing divergence times and the evolution of gene families in *D. chrysotoxum*. The green and red numbers represent the numbers of expanded gene families and contracted gene families, respectively. The blue portions of the pie charts show that the copy numbers of gene families are constant. Divergence times are represented by light blue bars at internodes; the divergence time is at a 95% confidence interval and shown by the range of these bars. The expansion and contraction of gene families are represented by members of branches (see Materials and methods and Supplementary Fig. 6). The orange part of the pie chart at the top left represents the ratio of 11,252 gene families found in the most recent common ancestor (MRCA) that expanded or contracted during recent differentiation events

A high-confidence phylogenetic tree was constructed, and the divergence times were estimated based on 274 single-copy genes from 17 different plant species (Supplementary Fig. 5 and Supplementary Table 6). As expected, *D. chrysotoxum* was sister to *D. catenatum*, forming an Epidendroideae clade together with *P. equestris*, *G. elata*, and *A. shenzhenica* located at the bases of Orchidaceae branches (Supplementary Fig. 6). The Orchidaceae divergence was estimated to have occurred 123 Mya; the divergence between subfamily Apostasioideae and subfamily Epidendroideae occurred 80 Mya; the divergence between *D. chrysotoxum* and *D. catenatum* occurred 11 Mya; and the divergence between *Dendrobium* and *Phalaenopsis* occurred 38 Mya (Fig. 3). Then, the expansion and contraction of orthologous gene families were analyzed. According to the results, 140 and 1112 gene families expanded and contracted, respectively, in the lineage leading to Orchidaceae. In *D. chrysotoxum*, 953 gene families were expanded, as opposed to 783 in *D. catenatum*, 853 in *P. equestris*, 358 in *G. elata*, and 562 in *A. shenzhenica*. At the same time, 1335 gene families were contracted in *D. chrysotoxum*, as opposed to 644 in *D. catenatum*, 1009 in *P. equestris*, 2748 in *G. elata*, and 1615 in *A. shenzhenica*. A greater number of expanded gene families in *D. chrysotoxum* may lead to a larger genome size than that in other sequenced orchid species^2,24,33,34^.

The ancestral clade of *Dendrobium* had 464 expanded gene families and 216 contracted gene families. The *D. chrysotoxum* clade had 953 expanded gene families and 1335 contracted gene families. In the ancestral clade of *Dendrobium*, there were 19 significantly expanded gene families, including 236 genes from *D. chrysotoxum*. In the *D. chrysotoxum* clade, 107 gene families were significantly expanded, including 1048 genes, and 43 gene families were significantly contracted, including 59 genes. We also conducted Gene Ontology (GO) enrichment analysis for the expanded gene families, and the GO terms “cytoplasmic part” and “intracellular organelle” were found to be enriched (Supplementary Table 13). In addition, the bidirectional sugar transporter gene *SWEET* was identified (Supplementary Fig. 7), whose product plays important roles in sugar translocation between compartments^41^, phloem loading for long-distance translocation^42^, pollen nutrition^43^, and seed filling^44^. Further phylogenetic analysis showed that 17 genes were expanded in clade II (Supplementary Fig. 7), suggesting that these *SWEET* genes might be associated with a fleshy stem that is abundant in polysaccharides and other medicinal compounds.

### Synteny analysis and whole-genome duplication (WGD)

**Fig. 4:**
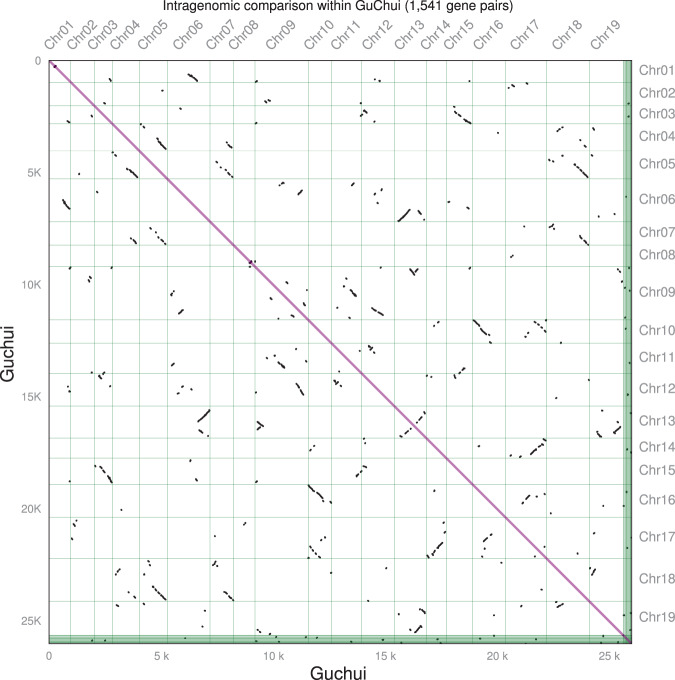
Self-collinearity map of *D. chrysotoxum* (Guchui). The values on the *X*- and *Y*-axes are the numbers of cumulative genes on the 19 chromosomes

Both the loss of a substantial fraction of genes and the increase in substitution rate complications were indicated by WGD in *D. chrysotoxum*, which is thought to have occurred among different orchid species^2^. WGD is evident in many lineages and is a practical method for genome expansion^45^. To determine the occurrence of WGDs in *D. chrysotoxum*, JCVI v0.9.14^46^ was used to analyze the protein sequences of *D. chrysotoxum*, *P. equestris*, *P. aphrodite*, and *D. catenatum* with the default parameters and obtain collinear gene pairs. There were 21,881 collinear gene pairs between *D. chrysotoxum* and *P. equestris*, 21,592 between *D. chrysotoxum* and *P. aphrodite*, 24,550 between *D. chrysotoxum* and *D. catenatum*, and 2800 between *D. chrysotoxum* and itself (Supplementary Table 14). Although *D. chrysotoxum* was assembled to the chromosome level, its self-collinearity was still very low compared to that of other sequenced orchid species. The collinearity between *D. catenatum* and *D. chrysotoxum* was fragmented, which may be the result of the quality of the *D. catenatum* genome, which was not at the chromosome level. The chromosomes of *Dendrobium* and *Phalaenopsis* showed a good corresponding relationship, indicating that after the divergence of *Dendrobium* and *Phalaenopsis*, the chromosomes were conserved, with few rearrangements. Syntenic figures show that the collinearity blocks were mainly in a 1:1 pattern, indicating that after the differentiation of *D. chrysotoxum*, no species-specific WGD events had occurred (Fig. 4; Supplementary Figs. 8–12).

**Fig. 5:**
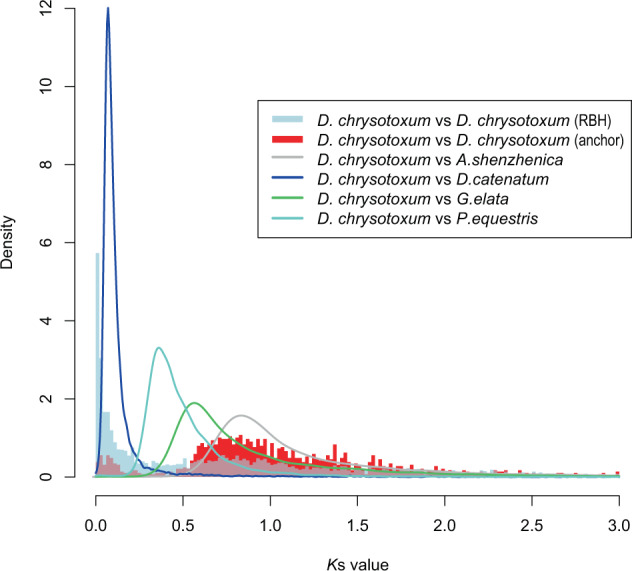
Distribution of *K*s values in the whole paranome of *D. chrysotoxum*. The *K*s distributions of paralogs using the reciprocal best hit (RBH) and anchor are shown as light blue and red histograms, respectively. The *K*s distribution for the one-to-one orthologs of *D. chrysotoxum*–*G. elata*, *D. chrysotoxum–A. shenzhenica*, *D. chrysotoxum*–*D. catenatum*, and *D. chrysotoxum*–*P. equestris* are shown as green, gray, blue, and cyan curves, respectively. RBH reciprocal best blast hit; “anchor” refers to colinear regions

The distributions of synonymous substitutions per synonymous site (*K*s) were estimated to infer polyploidization events that occurred in the *D. chrysotoxum* genome. There were two peaks in the distribution of *K*s for paralogous *D. chrysotoxum* genes: *K*s = 1.0 and 1.7–1.8 (Fig. 5). These results suggested that two polyploidization events occurred in *D. chrysotoxum*. To further verify the polyploidization events in *D. chrysotoxum*, its genome was compared with that of *P. equestris, A. shenzhenica*, and *D. catenatum*. The peaks in *K*s values between both *D. chrysotoxum/P. equestris* and *D. chrysotoxum/D. catenatum* were less than 1.0, suggesting that the events occurred before the differentiation of these three species. There was a diverging peak in the *K*s distribution of *D. chrysotoxum* and *A. shenzhenica* at *K*s = 0.7–0.8, which was smaller than but close to the *K*s peak of the Orchidaceae (*K*s = 1), indicating that extant orchid species differentiated immediately after experiencing a shared WGD event. Based on the evolution of gene families, species differentiation mainly occurred through gene loss with little gene expansion, which confirmed that the WGD event occurred in the most recent common ancestor of extant orchid species. The second peak in the *K*s distributions within *D. chrysotoxum* (1.7–1.8) indicated that the τ WGD had occurred in most monocot species^45^. Furthermore, the peak of the *K*s distribution in *D. chrysotoxum* was smaller than 0.2, suggesting that it originated from background (tandem) duplications and likely did not signify additional recent WGDs^2^. Therefore, this study found that *D. chrysotoxum* experienced two polyploidization events: an early WGD event was shared among all extant orchid species, and a later event that was shared among most monocot species.

### MADS-box genes and the evolution of flowers

MADS-box genes are among the most important regulators of plant floral development and compose major class of regulators mediating floral transition. In total, the *D. chrysotoxum* genome encodes 58 putative functional MADS-box genes and 1 pseudogene (Table 2; Supplementary Table 15). Interestingly, the number of MADS-box genes was similar to that in other sequenced orchid species but smaller than that in most sequenced angiosperms^2,24,33^. *D. chrysotoxum* has 31 type II MADS-box genes, which is higher than that found in *P. equestris* (29) and *A. shenzhenica* (27), but smaller than that of *D. catenatum* (35)^2,24,33^. Phylogenetic analysis (Supplementary Fig. 13) showed that, except for those in the MIKC*, *Bs*, and *OsMADS32* clades, most genes in the type II MADS-box clade were contracted. *Bs* genes are involved in the differentiation and development of ovules^47^. In *D. chrysotoxum*, there are four *Bs* members, more than the number found in other sequenced orchid species. The *Bs* genes had duplicated, as evidenced by higher seed production in *D. chrysotoxum* than in other sequenced orchid species. This must have been accompanied by duplication of the type I MADS-box gene Mα, as *D. chrysotoxum* has more Mα genes (19) than other sequenced orchid species (Table 2), ensuring seed development. In addition, there were no genes from the *FLOWERING LOCUS C* (*FLC*), *AGL12*, or *AGL15* clades in the *D. chrysotoxum* genome or other sequenced orchid genomes. In comparison with genes in the *AGL12* and *AGL15* clades, which are present in both rice and *Arabidopsis*, orthologous genes of *FLC*, *AGL12*, and *AGL15* might have been specifically lost in orchids. Although *AGL12*-like genes (*XAL1* in *A. thaliana*) are necessary for root development and flowering^48^, *D. chrysotoxum* and *P. equestris* have varying mechanisms that perform the same function^2^, showing that *D. chrysotoxum* is not a terrestrial orchid but is an epiphytic orchid.

**Table 2:** MADS-box genes in *D. chrysotoxum*, *A. shenzhenica*, *P. equestris*, *D. catenatum*, and *Arabidopsis thaliana*

Category	*P. equestris*^33^		*D. catenatum*^24^		D. chrysotoxum^*^		*A. shenzhenica*^2^		*A. thaliana*^37^	
	Functional	Pseudo	Functional	Pseudo	Functional	Pseudo	Functional	Pseudo	Functional	Pseudo
Type II (total)	29	1	35	11	31	0	27	4	45	5
MIKCc	28	1	32	9	28	0	25	3	43	4
MIKC^a^	1	0	3	2	4	0	2	1	2	0
Mδ	0	0	0	0	0	0	0	0	4	1
Type I (total)	22	8	28	1	26	1	9	0	62	36
Mα	10	6	15	1	19	1	5	0	20	23
Mβ	0	0	0	0	0	0	0	0	17	5
Mγ	12	2	13	0	8	1	4	0	21	8
Total	51	9	63	12	58	1	36	4	107	41

^a^This study

The *D. chrysotoxum* genome has 26 putative functional type I genes and 1 pseudogene (Table 2), which might have resulted in a lower expansion rate or a higher contraction rate compared with those of type II MADS-box genes in *D. chrysotoxum* (31 functional genes). Tandem gene duplication might play an important role in the increasing number of type I genes in the α group (type I Mα), suggesting that the type I genes have mainly been duplicated on a smaller scale from more-recent duplications^49^. Although members of the β group of type I MADS-box genes (type I Mβ) do exist in *A. thaliana*, poplar, and rice, they are absent in the *D. chrysotoxum* genome. Interactions among these type I MADS-box genes are essential for initiating endosperm development^50^; therefore, like in other sequenced orchids^2,24,33^, endosperm is also absent in *D. chrysotoxum*.

### Floral color regulatory pathway in *D. chrysotoxum*

**Fig. 6:**
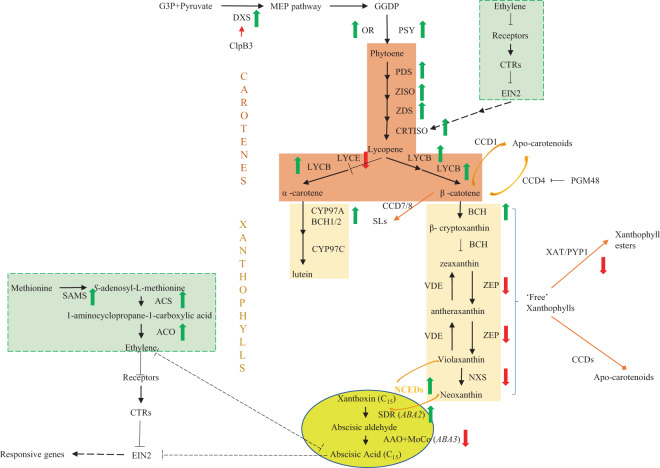
Gene expression patterns among the interactions between carotenoids, the ABA and ethylene biosynthesis pathways, and regulatory mechanisms in *D. chrysotoxum* (adapted from Watkins and Pogson^53^, Yin et al.^55^, Nisar et al.^101^, Sun et al.^102^, and Finkelstein^96^). Increased gene expression during the four developmental stages of flowers is shown by thick green arrows, while decreased gene expression is shown by thick red arrows. The enzymes or genes are indicated alongside the arrows. The dashed lines suggest that there are multiple steps. Carotenes are shown in orange, and light gold indicates xanthophylls. ABA-related genes are shown in yellow, and ethylene-related genes are shown in light green. CCDs (orange arrows) regulate carotenoid accumulation. Plastoglobule-localized metallopeptidase 48 (PGM48) is hypothesized to regulate CCD4. The orange protein regulates PSY activity, acting as a chaperone. ClpB3 (red arrow) regulates the activity of DXS, acting as an enzyme in the MEP pathway

The flowering time of a single flower of *D. chrysotoxum* was ~10 days^51,52^, the limit of which might be associated with yellow flower color. All photosynthetic tissues in each of the biological kingdoms can produce carotenoids^53^. More than 1100 naturally occurring carotenoids (http://carotenoiddb.jp/) are involved in many of the red, orange, and yellow colors of flowers^53^. These compounds also play important roles in photosynthesis. Interestingly, carotenoids function as precursors for the biosynthesis of abscisic acid (ABA)^53^. Moreover, ethylene plays a role in senescing flowers^54^. Ethylene and ABA regulate plant growth and development^55^ synergistically or antagonistically. We therefore analyzed the network involving carotenoid, ABA, and ethylene biosynthesis and regulation (Fig. 6).

Eighteen genes or gene family members in the carotenoid biosynthesis pathway and related regulatory mechanisms were identified (Supplementary Table 16). These genes encode phytoene synthase (PSY), orange protein, casein lytic proteinase B3 (ClpB3), deoxy-D-xylulose 5-phosphate synthase (DXS), phytoene desaturase, ζ-carotene isomerase, ζ-carotene desaturase, carotenoid isomerase (CRTISO), β-lycopene cyclase, ε-lycopene cyclase (LYCE), β-carotene hydroxylase, carotene ε-hydroxylase (CYP97C), zeaxanthin epoxidase (ZEP), violaxanthin de-epoxidase, neoxanthin synthase (NXS), xanthophyll acyl-transferase (XAT), plastoglobule-localized metallopeptidase 48, and carotenoid cleavage dioxygenase (CCD). The expression of these genes increased with flower development, except for *LYCE*, which is targeted for downregulation during biofortification, *ZEP*, *NXS*, and *XAT* (Supplementary Table 16; Fig. 6), suggesting that more carotenoids and fewer xanthophylls were produced during flowering to senescence.

The substrates used to produce ABA were neoxanthin and violaxanthin, and the process was regulated by nine-*cis*-epoxy carotenoid dioxygenases (NCEDs). The biosynthesis of ABA is catalyzed by the short-chain dehydrogenase/reductase-like (SDR1) enzyme abscisic aldehyde oxidase (AAO) and molybdenum cofactor (MoCo). The expression of *NCED* and *SDR* genes increased gradually with the development of flowers, while the expression of *AAO* and *MoCo* genes decreased gradually (Supplementary Table 17; Fig. 6). Furthermore, there were four *AAO* gene members detected in *Arabidopsis*, while there was only one gene detected in *D. chrysotoxum* (Supplementary Fig. 14). Taken together, these findings might indicate that relatively low amounts of ABA (C15) were produced, which might improve ethylene biogenesis.

For ethylene biogenesis, genes encoding three kinds of enzymes were identified. The expression of *Maker79017*, encoding S-AdoMet synthetase (SAMS), *Maker75695* and *Maker66290*, encoding ACC synthase (ACS), and *Maker29641*, encoding ACC oxidase (ACO), increased gradually, suggesting that increased amounts of ethylene were produced during the development of flowers (Supplementary Table 18; Fig. 6). *CONSTITUTIVE TRIPLE RESPONSE* 1 and *ETHYLENE INSENSITIVE 2* regulate the interaction between ethylene and the ABA pathway and are partially dependent on the MHZ5/CRTISO-mediated ABA pathway in rice^55^. Therefore, we also analyzed the expression patterns of the two genes in *D. chrysotoxum*, but there were no obvious differences in any of the four stages of flower development (Supplementary Table 18; Fig. 6).

In conclusion, carotenoid production increased gradually, and the content of xanthophylls decreased gradually in yellow *D. chrysotoxum* flowers during flowering to senescence. Less xanthophyll was degraded into less ABA, and less ABA led to more ethylene being produced. As a result, yellow flowers of *D. chrysotoxum* generally have a relatively short flowering period.

### Identification of the terpene synthases (TPS) and Hsp90 gene families and adaptive evolution

**Fig. 7:**
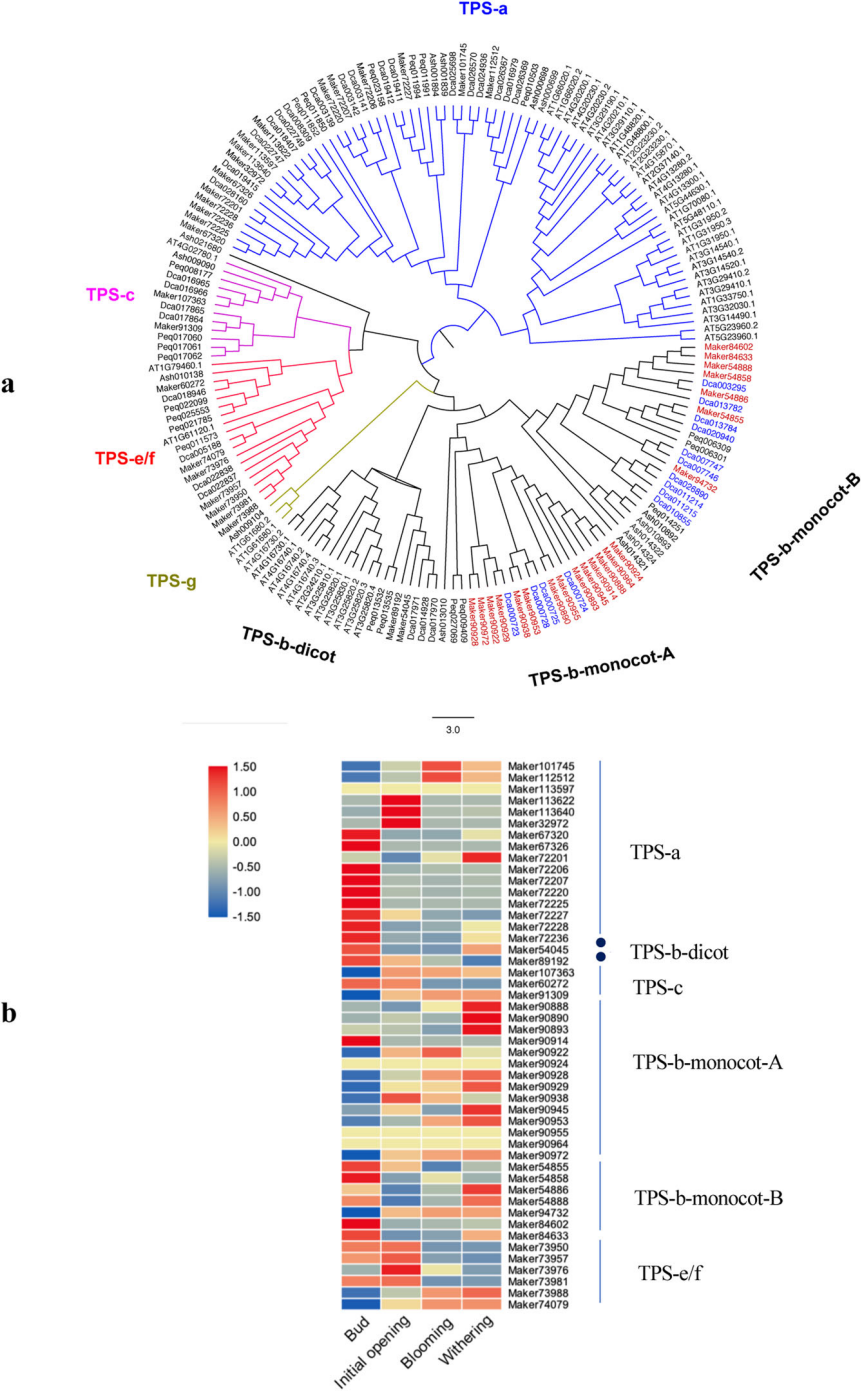
Analysis of TPS genes in *D. chrysotoxum*. **a** Phylogenetic analysis of TPS genes in *D. chrysotoxum*, *D. catenatum, A. shenzhenica, A. thaliana*, and *P. equestris*. Ash *A. shenzhenica*, Maker *D. chrysotoxum*, Dca *D. catenatum*, Peq *P. equestris*, AT *A. thaliana*. **b** Expression patterns of TPS genes in buds and in the initial flower opening, blooming, and withering stages of *D. chrysotoxum*

*Dendrobium* spp., with epiphytic or lithophytic lifestyles, frequently experience adverse environmental conditions, such as chilling and water deficit^56^. During plant responses to environmental stresses, volatile terpenes play critical roles^56^. Moreover, terpenes also play an important role in the formation of orchid floral scents^56^. TPSs are the key enzymes involved in terpene biosynthesis^57^. Different sizes of TPS families and subfamilies in plant species have evolved to synthesize a specific set of terpene compounds^57^. There are seven subfamilies in the TPS family: TPS-a, TPS-b, TPS-c, TPS-d, TPS-e/f, TPS-g, and TPS-h^57^. Among them, TPS-a encodes a sesquiterpene synthase that is found in both dicotyledonous and monocotyledonous plants. Angiosperm-specific TPS-b encodes a monoterpene synthase with an R(R)X8W motif that catalyzes the isomerization cyclization reaction. TPS-c belongs to the ancestral clade and catalyzes the activity of copalyl diphosphate synthase. Gymnosperm-specific TPS-d performs several functions, such as those of diterpene, monoterpene, and sesquiterpene synthases. TPS-e/f encodes copalyl diphosphate/kaurene synthases, which are critical enzymes for gibberellic acid production. Another angiosperm-specific TPS, TPS-g, encodes monoterpene synthase enzymes that lack the R(R)X8W motif. TPS-h has been observed only in *Selaginella moellendorffii*^58–61^. Phylogenetic analysis of the TPS gene family members and their expression in bud formation and initial flower opening, blooming, and withering are shown in Fig. 7. In this study, the TPS gene number in *D. chrysotoxum* was 48, which was greater than that in *D. catenatum* (42) (Fig. 7a). Moreover, there were 14 and 21 genes in *A. shenzhenica* and *P. equestris*, respectively. The *TPS-b* subfamily can be divided into monocot and eudicot clades. More *D. chrysotoxum* TPS genes than *D. catenatum* ones clustered in the monocot A clade—14 (red gene ID) and 4 (blue gene ID), respectively (Fig. 7a). Fewer TPS genes were found in *D. chrysotoxum* than in *D. catenatum* in the monocot B clade—7 (red gene ID) and 10 (blue gene ID), respectively (Fig. 7a). The different distribution patterns might contribute to the difference in terpenoid compositions between these two species, which needs further validation.

To explore heat stress-related genes in *D. chrysotoxum*, we also analyzed heat stress-related gene families across orchid species. Only two Hsp90 genes (red gene ID) were identified (clustering in group III), with high expression during bud formation (Supplementary Fig. 15a, b). This number was lower than that for the other four species (six were identified in *D. catenatum*, seven in *P. equestris*, six in *A. shenzhenica*, and seven in *A. thaliana*). This large gene loss might be related to resistance to heat stress.

## Conclusion

Although *D. chrysotoxum* has high ornamental and medicinal value, further molecular mechanism research and development of medicinal compounds have been limited by a lack of omics data. In this study, a chromosome-level reference genome of *D. chrysotoxum* with an assembled genome size of 1.37 Gb and 30,044 annotated protein-coding genes was obtained. *K*s analysis suggested that two polyploidization events occurred in *D. chrysotoxum*: a recent WGD shared among other orchid species and an ancient polyploidization event shared among most monocots (τ event). Phylogenetic analysis of the *SWEET* gene family in *D. chrysotoxum* showed that gene expansion occurred in clade II of the *SWEET* gene family, which might be related to fleshy stems containing an abundance of polysaccharides. Floral color regulation analysis showed that fewer xanthophylls degraded into ABA, which led to more ethylene production, thus accelerating the senescence of *D. chrysotoxum* flowers. The analysis of *D. chrysotoxum* helped elucidate the mechanism through which fleshy stems produce an abundance of polysaccharides and other medicinal compounds, as well as flowering time regulation, which is critical for industrial development. Our results provide the first high-quality genome of *Dendrobium* and give important insights into the molecular mechanism underlying the production of medicinal active ingredients, breeding, and orchid evolution.

## Materials and methods

### DNA preparation and sequencing

Fresh leaves of wild *D. chrysotoxum* were collected for genome sequencing. A modified cetyltrimethylammonium bromide protocol was used to extract the genomic DNA. To estimate genome size and heterozygosity, 143.78 Gb of raw data from paired-end libraries (PE150) constructed from a MGISEQ-2000 sequencer were generated. After data filtering was carried out by SOAPnuke v1.6.5 software with the parameters -n 0.02 -l 20 -q 0.4 -Q 2 -i -G --seqType 0 –rmdup, clean data (138.15 Gb) were obtained (Supplementary Table 1). Then, a SMRTbell Template Prep Kit 1.0 (PacBio, Menlo Park, CA, USA) and a PacBio Sequel system were used to construct and sequence the DNA libraries, respectively, for PacBio long-read sequencing. A total of 132.64 Gb of sequencing data (coverage of 96.12%) were generated, with an N50 read length of 19.5 kb (Supplementary Table 1). Furthermore, all libraries with a 500 bp insert size were sequenced on a NovaSeq platform (2 × 150 bp). We ultimately produced 169.25 Gb of data and 125.96 Gb of clean data for Hi-C analysis. The transcriptomes of flowers of *D. chrysotoxum* were obtained from Huang’s doctoral thesis^62^ to assist gene annotation.

### Genome assembly

Genome size and heterozygosity were measured using Jellyfish v.2.2.6 and GenomeScope (http://qb.cshl.edu/genomescope)^63^ based on a 17-K-mer distribution. Canu^64^ was used to assemble the PacBio sequencing reads, with the following parameters: minOverlapLength = 700; minReadLength = 1000; and corOutCoverage = 50. Then, Arrow software was used to polish the assembly, and Pilon v1.23^65^ was further used for correction of the assembly based on short reads, with the following parameters: fixed bases; mindepth 10; minqual 20; and diploid. Finally, the completeness and quality of the final assembled genome were evaluated with BUSCO v3^32^.

### Hi-C library construction and chromosome assembly

The raw reads produced by the NovaSeq sequencing platform were filtered by SOAPnuke^66^ (v1.6.5, https://github.com/BGI-flexlab/SOAPnuke) software with the following parameters: -n 0.02 -l 20 -q 0.4 -i –rmdup. Then, the obtained clean reads were compared with the preassembled contigs using Juicer^67^ software. After filtering the results and removing the misaligned reads, 3D-DNA^68^ software was used to preliminarily cluster, sequence, and direct the pseudochromosomes. Juicer-box was used to adjust, reset, and cluster the pseudochromosomes to improve the chromosome assembly quality. For the evaluation of the Hi-C assembly results, the final pseudochromosome assemblies were divided into 100 kb bins of equal lengths, and a heat map was used to visualize the interaction signals generated by the valid mapped read pairs between each bin.

### Genome annotation

Repetitive sequences are an important part of a genome and are divided into two types, namely, tandem repeats and interspersed repeats. Two methods, de novo prediction and homology-based searches, were used to annotate repeat sequences in the genome. RepeatMasker v4.0.7 and RepeatProteinMask v4.0.7 software^69^ (http://www.repeatmasker.org) were used to identify repetitive sequences based on the Repbase v21.12 database^69^ (http://www.girinst.org/repbase). For de novo prediction, a repetitive sequence database was constructed using RepeatModeler v1.0.8^70^ and LTR_FINDER v1.06^71^. RepeatMasker software and Tandem Repeats Finder v4.09^72^ were subsequently used to predict repeat sequences and identify tandem repeats in the genome, respectively. The annotation of high-quality protein-coding genes was carried out by integrating homology-based, de novo and transcriptome-based predictions. For homology-based prediction, protein sequences from six species (*Arabidopsis thaliana*, *Oryza sativa*, *Sorghum bicolor*, *Zea mays*, *G. elata*, and *P. equestris*) were used to align *D. chrysotoxum* genome sequences via Exonerate v2.2.0^73^. Then, the complete sequences of 3000 genes from the homology-based prediction method were used to produce a training model through Augustus v3.2.3^74^ and SNAP v2006-07-28^75^ software. The RNA-seq data of *D. chrysotoxum* were mapped to genome sequences through HISAT2 and StringTie software^76,77^. Finally, Maker v2.31.8^78^ was used to annotate and integrate the results generated by the above methods. BUSCO v3^32^ was then used to evaluate the completeness and quality of the gene models.

Functional annotation of the predicted gene models was carried out by BLAST v2.2.31^79^ software and aligned against the contents of the SwissProt^80^, TrEMBL (http://www.uniprot.org/), KEGG (http://www.genome.jp/kegg/), InterPro^81^, Nr, and GO (The Gene Ontology Consortium) databases^82^. For noncoding RNA annotations, tRNAscan-SE 1.3.1 (http://lowelab.ucsc.edu/tRNAscan-SE/)^83^ was used to annotate tRNA sequences. BLASTN^79^ was used to search for rRNA, and miRNA and snRNA sequences were predicted by Infernal 1.1 (http://infernal.janelia.org/) software^84^.

### WGD analysis

*K*s distribution analysis was used to infer the occurrence of WGD events in *D. chrysotoxum* and those between *D. chrysotoxum* and *A. shenzhenica*, *D. catenatum*, and *P. equestris*. BLASTP^79^ was used to search for putative paralogous and orthologous genes within and between genomes by alignment of each genome pair. MCScanX v1.5.2^85^ was used to identify colinear regions, and then CodeML in the PAML package^86^ was used to calculate the *K*s value of each salicoid duplicated gene pair. We used CAFÉ^87^ to evaluate the significance of each expanded and contracted gene family (*P* < 0.01).

### SWEET gene family analyses

To identify SWEET proteins, proteomic datasets of four orchid species (*D. chrysotoxum*, *A. shenzhenica*, *D. catenatum*, and *P. equestris*) and *A. thaliana* were constructed. The MtN3_slv domain PF03083 model profile from the Pfam database^88^ was used for performing local searches of proteome datasets containing five species via the HMMER program^89^. The SWEET protein sequences were aligned with MAFFT^90^. The alignment was then used for phylogenetic tree reconstruction by PhyML 3.0^91–93^ with the default parameters.

### MADS-box gene family analysis

The sequences of the MADS-box proteins of *A. thaliana* and the HMM profile (PF00319) were obtained from the Arabidopsis information resource (TAIR) (https://www.arabidopsis.org/) and the Pfam database^88^, respectively. Then, the sequences of the MADS-box gene family members in the *D. chrysotoxum* genome were obtained using HMMER 3.2.1 software^89^ and BLASTP^83^ methods. The obtained amino acid sequences were used for TBLASTN^79^ analysis of the *D. chrysotoxum* transcriptomic assemblies. SMART^94^ was subsequently used to confirm the obtained sequences by domain analysis. MEGA X^95^ was then used for the alignment of the candidate genes, and the CIPRES website (https://www.phylo.org/portal2/) was used for phylogenetic tree construction. iTOL (https://itol.embl.de) was subsequently used to visualize the phylogenetic trees.

### Identification of genes involved in the carotenoid, ABA, and ethylene biosynthesis pathways and regulatory mechanisms in *D. chrysotoxum*

The sequences of all 17 genes or gene family members involved in the carotenoid biosynthesis pathway and regulatory mechanisms in *A. thaliana, Triticum aestivum*, and *Pantoea ananatis*^53^ were used as queries to search against the protein database of *D. chrysotoxum*. The obtained amino acid sequences were aligned using MAFFT^90^. We then manually inspected the aligned sequences and removed any obviously inconsistent sequences.

Four genes or gene family members involved in the ABA biosynthesis pathway or regulatory mechanisms in *Arabidopsis* were obtained^96^. BLASTP^79^ was used to search for homologous genes by querying the protein database of *D. chrysotoxum*. After aligning the amino acid sequences with MAFFT^90^ software, we removed any obviously inconsistent sequences.

The sequences of genes encoding SAMS, ACS, and ACO, all of which are involved in the ethylene biosynthesis pathway, in *Arabidopsis*^97^ were used as queries for searching proteins by BLASTP^79^ software.

For gene families, a phylogenetic tree was constructed with PhyML^98^ based on the alignment of sequences from *D. chrysotoxum*, *D. catenatum*, *A. shenzhenica*, *P. equestris*, and *A. thaliana*. The tree was generated by the maximum likelihood method based on the Jones–Taylor–Thornton (JTT) matrix-based model^99^, and the fast likelihood-based method was used for phylogenetic tests with SH-like branch supports.

### Gene expression analysis

Transcriptome data from flowers at four developmental stages (flower buds, initial flowering stage, blooming period, and withering flowers), stems, and leaves were obtained (BioProject PRJNA691441), and Salmon v1.3.0^100^ was used to quantify gene expression, with the default settings.

### TPS and Hsp90 gene family identification

The HMM profiles for PF01397 (Terpene_synth) and PF03936 (Terpene_synth_C) were downloaded from the Pfam database (pfam.xfam.org/), and both profiles were used to carry out HMM searches against the information of the protein databases for five species (*D. chrysotoxum, Dendrobium catenatum, P. equestris, Apostasia shenzhenica*, and *A. thaliana*). The sequences aligned with MAFFT^90^ were used for phylogenetic tree construction through PhyML^79^. The tree was generated by the maximum likelihood method based on the JTT matrix-based model^99^ and the bootstrap method for phylogenetic tests with 1000 replications. Similarly, the HMM profile for PF00183 (Hsp90) was downloaded from the Pfam database (pfam.xfam.org/), and the subsequent steps were the same as those for TPS gene family identification.

## Supplementary information

Supplementary Information

Supplementary Table 13

## Data Availability

All the data from this study have been deposited in the NCBI database under BioProject ID PRJNA664445.
