# Supplementary material for: Chromosome-scale assembly of the Dendrobium chrysotoxum genome enhances the understanding of orchid evolution
Source: Hortic Res. 2021 Sep 1;8:183. doi: 10.1038/s41438-021-00621-z (PMC8408244; doi:10.1038/s41438-021-00621-z)
Supplement: Supplementary file 1 — Supplementary Information [file 41438_2021_621_MOESM1_ESM.docx]

**Supplementary Information**

**Chromosome-scale assembly of the *Dendrobium chrysotoxum* genome enhances the understanding of orchid evolution**

Yongxia Zhang^1^, Guo-Qiang Zhang^2,3^, Diyang Zhang^4^, Xue-Die Liu^4,5^, Xin-Yu Xu^4^, Wei-Hong Sun^4,5^, Xia Yu^4^, Xiaoen Zhu^1^, Zhi-Wen Wang^9^, Xiang Zhao^9^, Wen-Ying Zhong^9^, Hongfeng Chen^6^, Wei-Lun Yin^4,8^, Tengbo Huang^1^*, Shan-Ce Niu^7^*, Zhong-Jian Liu^4^*

**Running title:** *Dendrobium chrysotoxum* genome

Content

[Supplementary Figures 4](#_Toc59631187)

[Supplementary Figure 1. Genome size and heterozygosity estimation using 17 *K*-*mer* distribution. 4](#_Toc59631188)

[Supplementary Figure 2. Gene structure prediction statistical results. *D. chrysotoxum* compared with the genetic elements of related species. Window refers to the length represented by each point on the horizonal coordinate. 5](#_Toc59631189)

[Supplementary Figure 3. Sequence divergence rate of four different TEs using RepeatMasker annotation. 6](#_Toc59631190)

[Supplementary Figure 4. Sequence divergence rate of four different TEs using *de novo* annotation. 7](#_Toc59631191)

[Supplementary Figure 5. Classification and distribution of homologous gene families among species. 8](#_Toc59631192)

[Supplementary Figure 6. Phylogenetic tree constructed based on the filtered single-copy gene family; each branch length represents the neutral evolution rate. 9](#_Toc59631193)

[Supplementary Figure 7. Phylogenetic analysis of SWEET genes from *A. thaliana*, *D. chrysotoxum*, *P. equestris*, *A. shenzhenica,* and *D. catenatum*. 10](#_Toc59631194)

[Supplementary Figure 8. Collinear map of *D. chrysotoxum* (GuChui) and *D. catenatum* (DCAT). 11](#_Toc59631195)

[Supplementary Figure 9. Syntenic depths map of *D. chrysotoxum* (GuChui) and *D. catenatum* (DCAT). 12](#_Toc59631196)

[Supplementary Figure 10. Collinear diagram of *D. chrysotoxum* (GuChui) and *P. aphrodite* (PAPH)*.* 13](#_Toc59631197)

[Supplementary Figure 11. Collinear depth distribution map of *D. chrysotoxum* (GuChui) and *P. aphrodite* (PAPH)*.* 14](#_Toc59631198)

[Supplementary Figure 12. *Ks* distribution identified by different methods.. 15](#_Toc59631199)

[Supplementary Figure 13. Phylogenetic analysis of MADS-box genes from *D. chrysotoxum*, *Arabidopsis thaliana* and *Oryza sativa*. Maker, *D. chrysotoxum*. 16](#_Toc59631200)

[Supplementary Figure 14. Phylogeny tree of AAO from *Dendrobium chrysotoxum, Dendrobium catenatum, Apostasia shenzhenica, Phalaenopsis equestris,* and *Arabidopsis thaliana* constructed by the PhyML with the Jones–Taylor–Thornton (JTT) matrix-based model. 17](#_Toc59631201)

[Supplementary Figure 15. Analysis of *D. chrysotoxum* Hsp90 genes. 18](#_Toc59631202)

[Supplementary Tables 19](#_Toc59631203)

[Supplementary Table 1. Summary of DNA sequencing data. 19](#_Toc59631204)

[Supplementary Table 2. Summary of Pacbio assembly results of *D. chrysotoxum*. 20](#_Toc59631205)

[Supplementary Table 3. BUSCO assessment of the *D. chrysotoxum* and *P. aphrodite* genomes’ assembly. 21](#_Toc59631206)

[Supplementary Table 4. Chromosome length by Hi-C assembly. 22](#_Toc59631207)

[Supplementary Table 5. Summary of HiC-assembly results. 23](#_Toc59631208)

[Supplementary Table 6. Summary of orthologous gene families in 17 sequenced plant species. 24](#_Toc59631209)

[Supplementary Table 7. BUSCO assessment of the *D. chrysotoxum* and *P. aphrodite* genomes’ annotation. 25](#_Toc59631210)

[Supplementary Table 8. Prediction of gene structures in *D. chrysotoxum*. 26](#_Toc59631211)

[Supplementary Table 9. Statistics of non-coding RNA in the *D. chrysotoxum* genome. 27](#_Toc59631212)

[Supplementary Table 10. Statistical results of repeat sequences. 28](#_Toc59631213)

[Supplementary Table 11. Statistics of repeat sequences in *D. chrysotoxum*. 29](#_Toc59631214)

[Supplementary Table 12. Statistical results of functional annotation. 30](#_Toc59631215)

[Supplementary Table 13. Gene Ontology (GO) enrichment results of significantly expanded gene families in *D. chrysotoxum* (see separate files). 31](#_Toc59631216)

[Supplementary Table 14. Statistical results of whole gene collinearity analysis. 32](#_Toc59631217)

[Supplementary Table 15. List of MADS-box genes identified in *D. chrysotoxum*. 33](#_Toc59631218)

[Supplementary Table 16. The expression of genes in carotenoid biosynthesis pathway and regulatory mechanisms. 35](#_Toc59631219)

[Supplementary Table 17. The expression of gene in abscisic acid biosynthesis pathway. 36](#_Toc59631220)

[Supplementary Table 18. The expression of genes in Ethylene biosynthesis pathway and regulatory mechanisms. 37](#_Toc59631221)

# Supplementary Figures


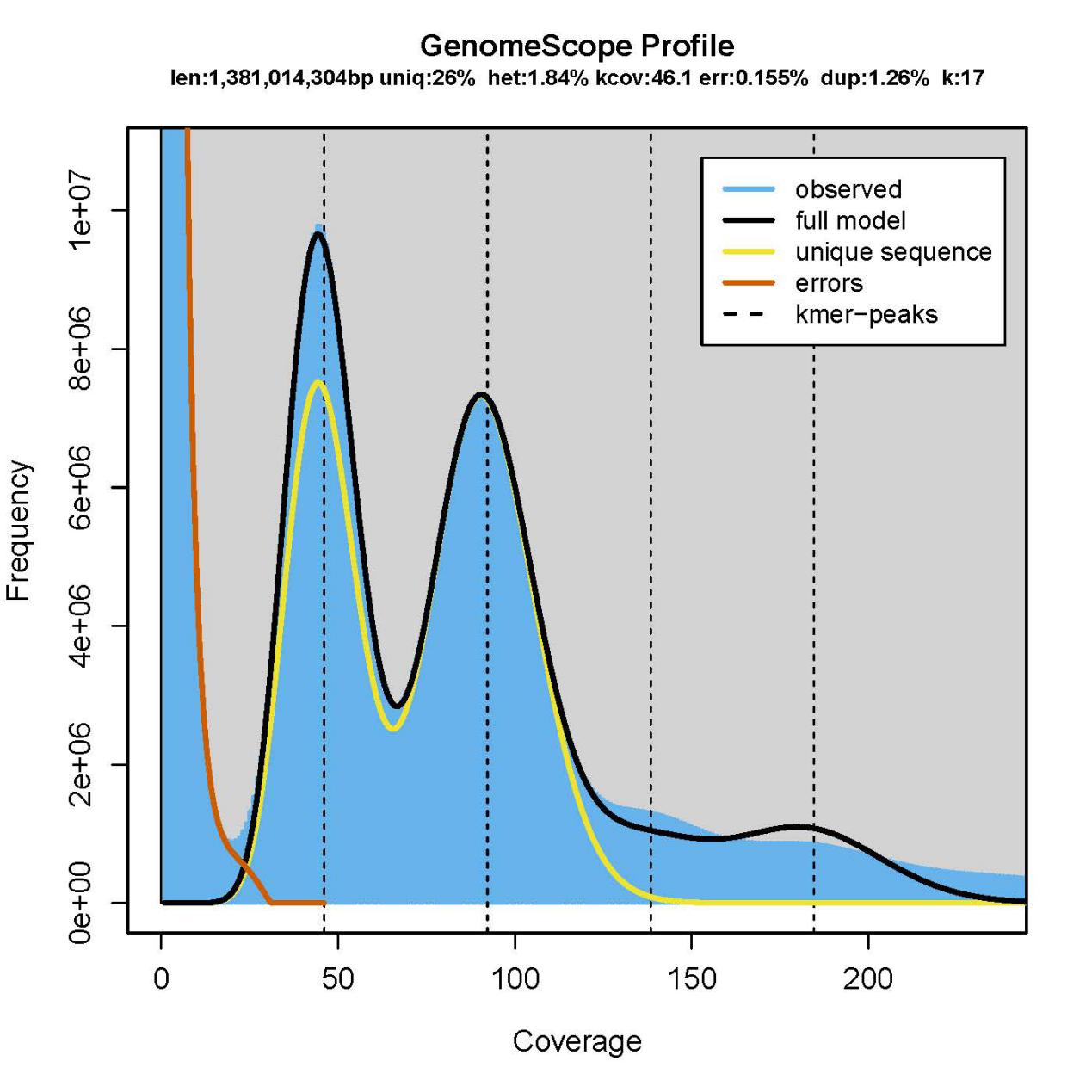


## Supplementary Figure 1. Genome size and heterozygosity estimation using 17 *K*-*mer* distribution.

## Supplementary Figure 2. Gene structure prediction statistical results. *D. chrysotoxum* compared with the genetic elements of related species. Window refers to the length represented by each point on the horizonal coordinate.


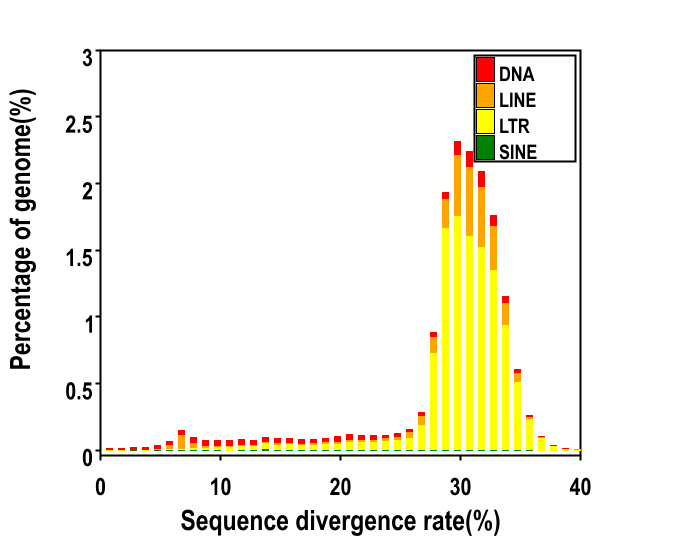


## Supplementary Figure 3. Sequence divergence rate of four different TEs using RepeatMasker annotation.


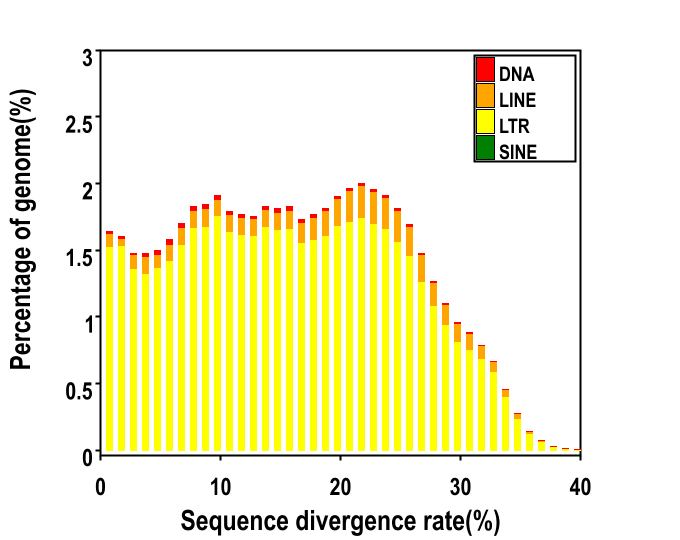


## Supplementary Figure 4. Sequence divergence rate of four different TEs using *de novo* annotation.


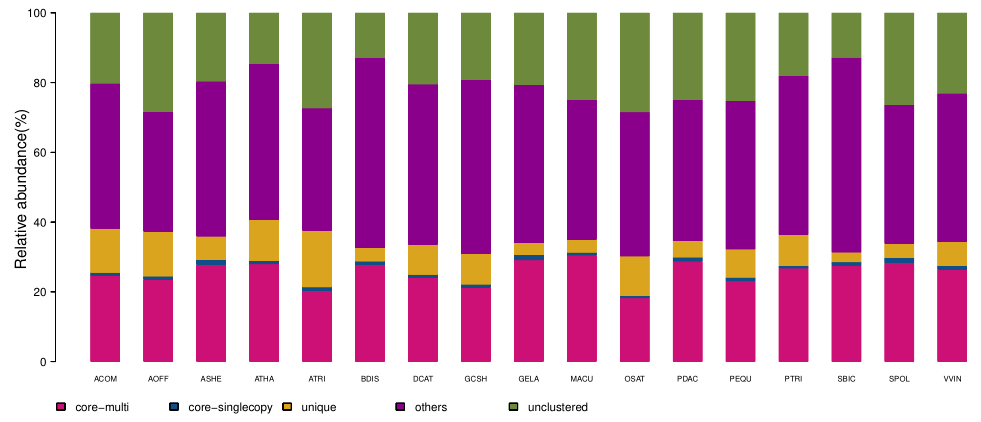


## Supplementary Figure 5. Classification and distribution of homologous gene families among species.

## Supplementary Figure 6. Phylogenetic tree constructed based on the filtered single-copy gene family; each branch length represents the neutral evolution rate.

## Supplementary Figure 7. Phylogenetic analysis of SWEET genes from *A. thaliana*, *D. chrysotoxum*, *P. equestris*, *A. shenzhenica,* and *D. catenatum*. Red gene IDs are expanded genes. Ash, *A. shenzhenica*; Maker, *D. chrysotoxum*; Dca, *D. catenatum*; Peq, *P. equestris*; AT, *A. thaliana*.

## Supplementary Figure 8. Collinear map of *D. chrysotoxum* (Guchui) and *D. catenatum* (DCAT).

## Supplementary Figure 9. Syntenic depths map of *D. chrysotoxum* (Guchui) and *D. catenatum* (DCAT).

## Supplementary Figure 10. Collinear diagram of *D. chrysotoxum* (Guchui) and *P. aphrodite* (PAPH)*.*

## Supplementary Figure 11. Collinear depth distribution map of *D. chrysotoxum* (Guchui) and *P. aphrodite* (PAPH)*.*

**a**


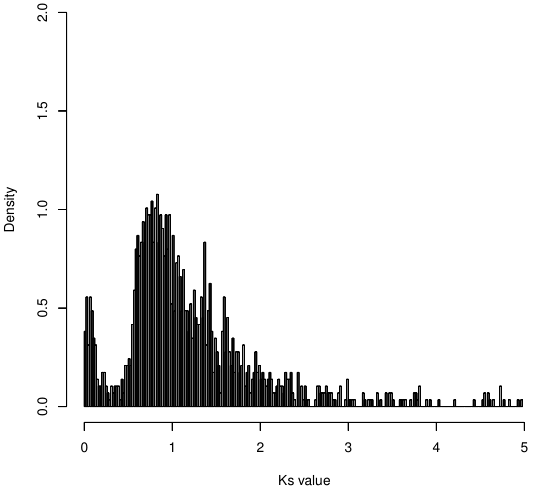

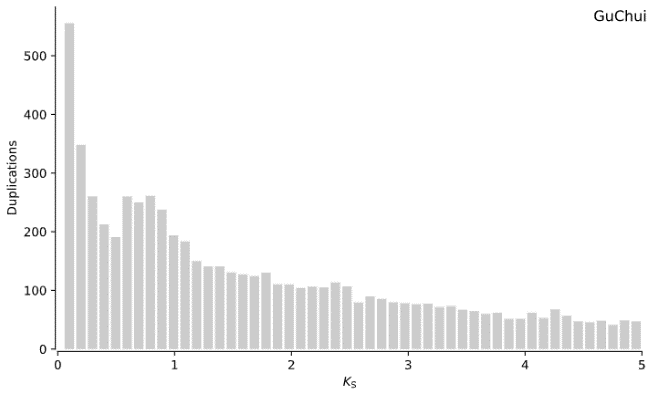

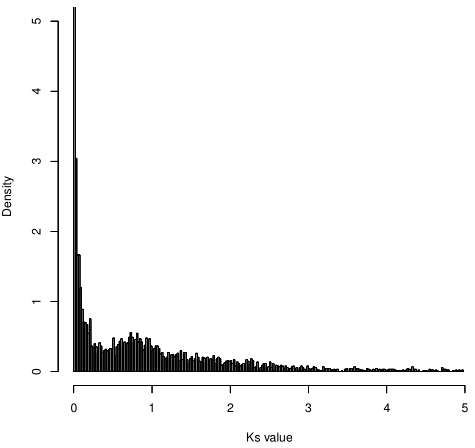


**b**

**c**

## Supplementary Figure 12. *Ks* distribution identified by different methods. a. *Ks* distribution of RBH gene pairs in *D. chrysotoxum*. b. *Ks* distribution identified by WGD software in *D. chrysotoxum*. c. *Ks* distribution of homologous genes in a collinear block of *D. chrysotoxum*. The distribution of *Ks* identified by the above different methods was consistent, and the peak value of *Ks* was at 0.8, which is consistent with the sequenced orchids in previous studies and represents a WGD event experienced by the common ancestor of orchids. The results also support that the earlier the differentiation, the larger the *Ks* peak.

**
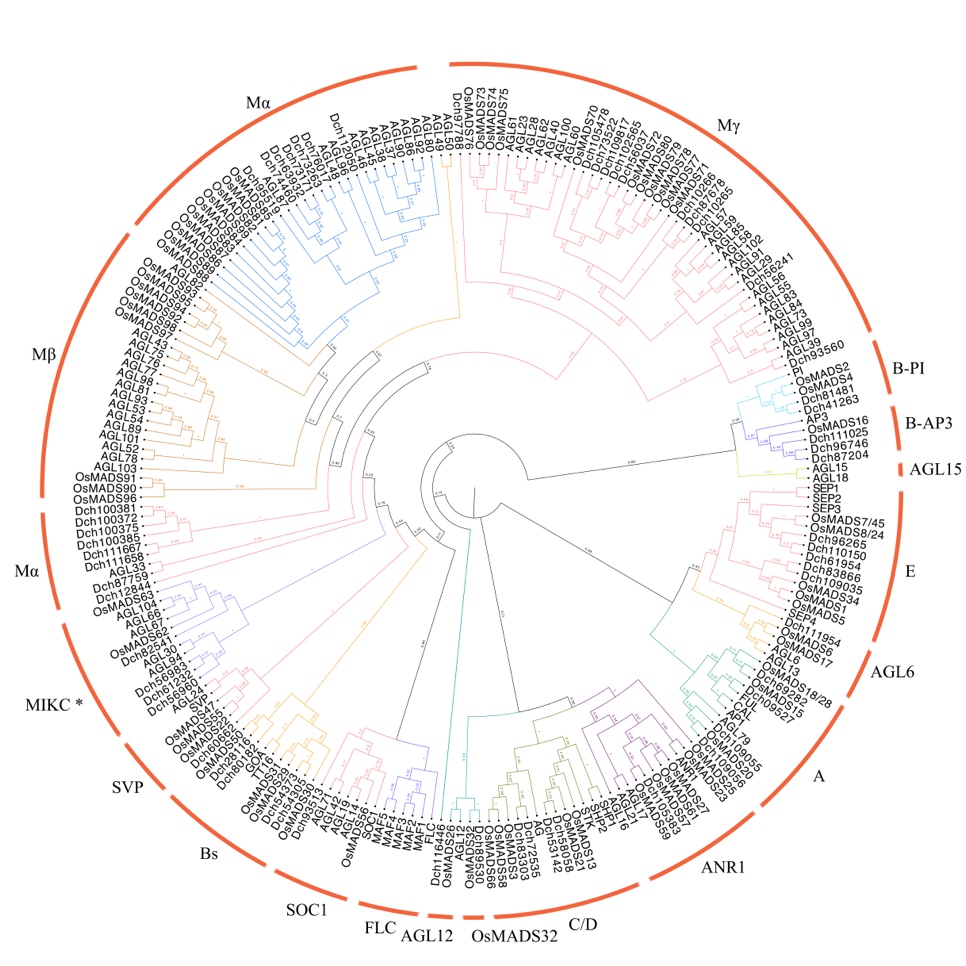
**

## Supplementary Figure 13. Phylogenetic analysis of MADS-box genes from *D. chrysotoxum*, *Arabidopsis thaliana* and *Oryza sativa*. Dch, *D. chrysotoxum*.

## Supplementary Figure 14. Phylogeny tree of AAO from *Dendrobium chrysotoxum, Dendrobium catenatum, Apostasia shenzhenica, Phalaenopsis equestris,* and *Arabidopsis thaliana* constructed by the PhyML with the Jones–Taylor–Thornton (JTT) matrix-based model. Blue branch showed the AAO clade; red font is the *AAO* gene from *Dendrobium chrysotoxum;* blue font indicated *Arabidopsis AAO* genes.


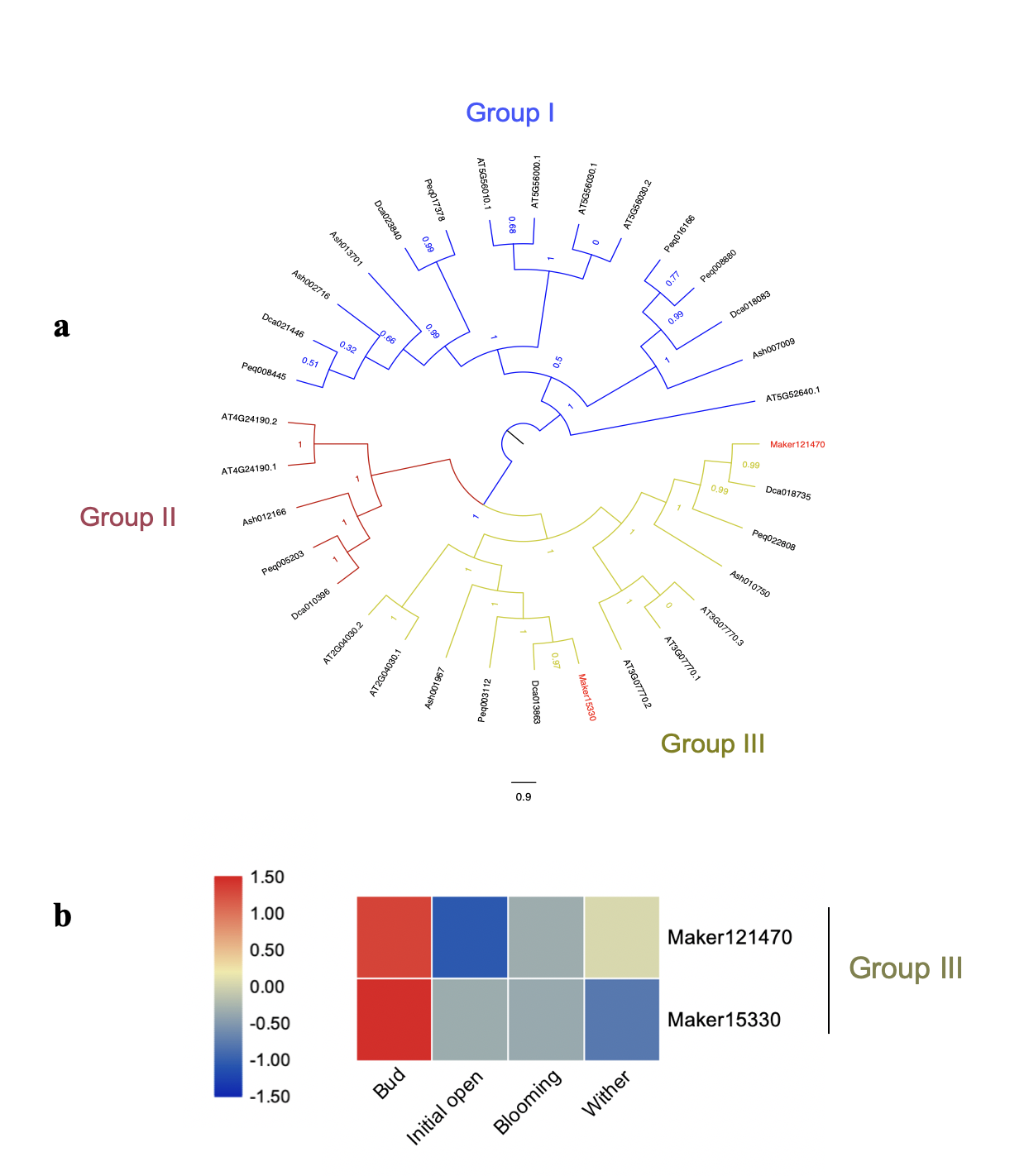


## Supplementary Figure 15. Analysis of *D. chrysotoxum* Hsp90 genes. a. Phylogenetic relationships of *D. chrysotoxum*, *D. catenatum,* *A. shenzhenica, A. thaliana* and *P. equestris* Hsp90 proteins. Ash, *A. shenzhenica*; Maker, *D. chrysotoxum*; Dca, *D. catenatum*; Peq, *P. equestris*; AT, *A. thaliana*. b. Expression patterns of Hsp90 in bud, initial open flower, blooming flower and wither flower of *D. chrysotoxum*.

# Supplementary Tables

## Supplementary Table 1. Summary of DNA sequencing data.

| **Sequencing platform** | **Read type** | **Clean Data (Gb)/ subreads** | **Insert size (bp)** | **Total data (Gb)** | **Read length / Mean subread length (N50) (bp)** | **Sequence coverage**^1^ **(X)** |
| --- | --- | --- | --- | --- | --- | --- |
| MGISEQ-2000 | Illumina pair-end reads | 138.15 | 500 | 143.78 | 2 x 150 | 104.19 |
| NovaSeq | Hi-C pair-end reads | 125.96 | 500 | 169.25 | 2 x 150 | 122.64 |
| PacBio Sequel | PacBio subreads | 7,136,946 | - | 132.64 | 12,942.98 (19,505) | 96.12 |

^1^Depth was calculated under the estimate of a genome size of 1.38 Gb.

## Supplementary Table 2. Summary of Pacbio assembly results of *D. chrysotoxum*.

| **Contig** | | | | |
| --- | --- | --- | --- | --- |
| **Size (bp)** | | | **Number** | |
| N90 | 411,893 | | | 898 |
| N80 | 699,493 | | | 646 |
| N70 | 988,173 | | | 486 |
| N60 | 1,234,715 | | | 364 |
| N50 | 1,540,953 | | | 265 |
| Longest | 8,657,479 | | | - |
| Total Length | 1,367,445,997 | | | - |
| Total Number (>=100 bp) | - | | | 2,302 |
| Total Number (>=2000 bp) | - | | | 2,291 |
| GC Rate | | 0.355 | | |

## Supplementary Table 3. BUSCO assessment of the *D. chrysotoxum* and *P. aphrodite* genomes’ assembly.

| **Species** | **Type** | **Number** | **Percentage** |  |
| --- | --- | --- | --- | --- |
|  | Complete BUSCOs(C) | 1,242 | 90.33% |  |
|  | Complete and single-copy BUSCOs (S) | 1,218 | 88.58% |  |
|  | Complete and duplicated BUSCOs (D) | 24 | 1.75% |  |
| *P. aphrodite* | Fragmented BUSCOs (F) | 35 | 2.55% | |
|  | Missing BUSCOs (M) | 98 | 7.13% |  |
|  | Total BUSCO groups searched | 1,375 | - |  |
|  |  |  |  |  |
|  | Complete BUSCOs(C) | 1,242 | 90.33% |  |
|  | Complete and single-copy BUSCOs (S) | 1,125 | 81.82% |  |
| *D. chrysotoxum* | Complete and duplicated BUSCOs (D) | 117 | 8.51% |  |
|  | Fragmented BUSCOs (F) | 49 | 3.56% |  |
|  | Missing BUSCOs (M) | 84 | 6.11% |  |
|  | Total BUSCO groups searched | 1,375 | - |  |

## Supplementary Table 4. Chromosome length by Hi-C assembly.

| **Chromosome ID** | **Contig number** | **Length(bp)** | **Length ratio** |
| --- | --- | --- | --- |
| Chr01 | 75 | 58,699,466 | 4.29% |
| Chr02 | 57 | 60,674,785 | 4.43% |
| Chr03 | 56 | 38,283,235 | 2.80% |
| Chr04 | 102 | 81,914,847 | 5.99% |
| Chr05 | 73 | 60,425,906 | 4.42% |
| Chr06 | 117 | 98,428,665 | 7.19% |
| Chr07 | 76 | 57,422,580 | 4.20% |
| Chr08 | 83 | 45,230,344 | 3.31% |
| Chr09 | 99 | 98,780,041 | 7.22% |
| Chr10 | 50 | 53,244,469 | 3.89% |
| Chr11 | 114 | 67,798,029 | 4.96% |
| Chr12 | 63 | 60,234,544 | 4.40% |
| Chr13 | 62 | 60,242,052 | 4.40% |
| Chr14 | 65 | 47,375,978 | 3.46% |
| Chr15 | 39 | 46,988,260 | 3.43% |
| Chr16 | 99 | 87,905,214 | 6.42% |
| Chr17 | 94 | 94,615,362 | 6.92% |
| Chr18 | 82 | 100,493,053 | 7.34% |
| Chr19 | 93 | 91,318,610 | 6.67% |
| UN | 805 | 58111057 | 4.25% |

## Supplementary Table 5. Summary of HiC-assembly results.

| **Contig** | | | | **Scaffold** | | | |
| --- | --- | --- | --- | --- | --- | --- | --- |
| **Size(bp)** | | **Number** | | **Size(bp)** | | **Number** | |
| N90 | 411,893 | | 898 | | 45,230,344 | | 18 |
| N80 | 696,269 | | 647 | | 53,244,469 | | 15 |
| N70 | 984,844 | | 486 | | 60,234,544 | | 12 |
| N60 | 1,234,715 | | 364 | | 60,425,906 | | 10 |
| N50 | 1,540,953 | | 265 | | 67,798,029 | | 8 |
| Longest | 8,657,479 | | - | | 100,493,053 | | - |
| Total Length | 1,367,445,997 | | - | | 1,310,075,440 | | - |
| Total Number (>=100 bp) | - | | 2304 | | - | | 823 |

## Supplementary Table 6. Summary of orthologous gene families in 17 sequenced plant species.

| **Species** | **Genes** | **Unclustered genes** | **Clustered genes** | **Families** | **Unique families** | **Unique family genes** | **Common families** | **Common family genes** | **Single copy** | **Average genes per family** |
| --- | --- | --- | --- | --- | --- | --- | --- | --- | --- | --- |
| *A. comosus* | 27,024 | 5,462 | 21,562 | 12,942 | 787 | 3,408 | 3,785 | 6,907 | 274 | 1.666 |
| *A. officinalis* | 27,375 | 7,773 | 19,602 | 11,940 | 836 | 3,512 | 3,785 | 6,699 | 274 | 1.642 |
| *A. shenzhenica* | 20,560 | 4,032 | 16,528 | 11,808 | 335 | 1,390 | 3,785 | 5,995 | 274 | 1.4 |
| *A. thaliana* | 27,416 | 3,993 | 23,423 | 12,653 | 781 | 3,212 | 3,785 | 7,938 | 274 | 1.851 |
| *A. trichopoda* | 25,933 | 7,083 | 18,850 | 12,238 | 985 | 4,210 | 3,785 | 5,538 | 274 | 1.54 |
| *B. distachyon* | 26,415 | 3,402 | 23,013 | 14,917 | 352 | 1,014 | 3,785 | 7,600 | 274 | 1.543 |
| *D. catenatum* | 26,791 | 5,483 | 21,308 | 14,092 | 578 | 2,297 | 3,785 | 6,695 | 274 | 1.512 |
| *D. chrysotoxum* | 30,044 | 5,765 | 24,280 | 13,324 | 606 | 2,633 | 3,785 | 6,657 | 274 | 1.822 |
| *G. elata* | 18,019 | 3,715 | 14,304 | 10,545 | 229 | 610 | 3,785 | 5,521 | 274 | 1.356 |
| *M. acuminata* | 34,241 | 8,517 | 25,724 | 12,540 | 486 | 1,242 | 3,785 | 10,721 | 274 | 2.051 |
| *O. sativa* | 42,189 | 11,999 | 30,190 | 16,547 | 1,265 | 4,787 | 3,785 | 7,959 | 274 | 1.824 |
| *P. dactylifera* | 23,890 | 5,972 | 17,918 | 10,826 | 369 | 1,115 | 3,785 | 7,146 | 274 | 1.655 |
| *P. equestris* | 26,471 | 6,679 | 19,792 | 13,495 | 636 | 2,142 | 3,785 | 6,388 | 274 | 1.467 |
| *P. trichocarpa* | 40,984 | 7,407 | 33,577 | 14,108 | 1,177 | 3,676 | 3,785 | 11,253 | 274 | 2.38 |
| *S. bicolor* | 27,160 | 3,473 | 23,687 | 15,312 | 294 | 767 | 3,785 | 7,749 | 274 | 1.547 |
| *S. polyrhiza* | 18,357 | 4,841 | 13,516 | 10,064 | 248 | 741 | 3,785 | 5,475 | 274 | 1.343 |
| *V. vinifera* | 25,328 | 5,847 | 19,481 | 12,576 | 595 | 1,736 | 3,785 | 6,956 | 274 | 1.549 |

Note: Unclustered genes refers to the number of genes unique to the species; Unique families refers to gene families unique to the species.

## Supplementary Table 7. BUSCO assessment of the *D. chrysotoxum* and *P. aphrodite* genomes’ annotation.

| **Species** | **Type** | **Number** | **Percentage** |
| --- | --- | --- | --- |
|  | Complete BUSCOs(C) | 1,297 | 94.32% |
|  | Complete and single-copy BUSCOs (S) | 1,274 | 92.65% |
|  | Complete and duplicated BUSCOs (D) | 23 | 1.67% |
| *P. aphrodite* | Fragmented BUSCOs (F) | 28 | 2.04% |
|  | Missing BUSCOs (M) | 50 | 3.64% |
|  | Total BUSCO groups searched | 1,375 | - |
|  |  |  |  |
|  | Complete BUSCOs(C) | 1315 | 95.64% |
|  | Complete and single-copy BUSCOs (S) | 1235 | 89.82% |
| *D. chrysotoxum* | Complete and duplicated BUSCOs (D) | 80 | 5.82% |
|  | Fragmented BUSCOs (F) | 20 | 1.45% |
|  | Missing BUSCOs (M) | 40 | 2.91% |
|  | Total BUSCO groups searched | 1,375 | - |

## Supplementary Table 8. Prediction of gene structures in *D. chrysotoxum*.

|  | **Gene set** | **Number** | **Average** | **Average** | **Average** | **Average** | **Average** |
| --- | --- | --- | --- | --- | --- | --- | --- |
|  |  |  | **mRNA** | **CDS** | **exon per** | **exon** | **intron** |
|  |  |  | **length (bp)** | **length (bp)** | **gene** | **length (bp)** | **length (bp)** |
| Denovo | Augustus | 49,763 | 5281.51 | 839.03 | 2.83 | 296.77 | 2431.3 |
|  | SNAP | 145,612 | 5085.98 | 490.97 | 2.88 | 170.63 | 2447.5 |
| Homolog | *Arabidopsis thaliana* | 59,398 | 6078.15 | 881.47 | 3.22 | 273.92 | 2342.99 |
|  | *Oryza sativa* | 70,498 | 5649.71 | 850.58 | 3.02 | 281.96 | 2379.77 |
|  | *Sorghum bicolor* | 62,870 | 6219.25 | 891.2 | 3.27 | 272.95 | 2352.28 |
|  | *Zea mays* | 85,147 | 4872.26 | 715.26 | 2.82 | 253.26 | 2278.73 |
|  | *Gastrodia elata* | 54,950 | 5920.87 | 836.66 | 3.16 | 265.11 | 2358.35 |
|  | *Phalaenopsis equestris* | 136,048 | 3443.8 | 606.14 | 2.29 | 265.05 | 2205.14 |
| RNA-seq | -- | 33387 | 16066.78 | 1667.93 | 5.32 | 313.62 | 3334.38 |
| Maker | -- | 30,044 | 10947.05 | 1033.86 | 4.5 | 229.98 | 2642.87 |

## Supplementary Table 9. Statistics of non-coding RNA in the *D. chrysotoxum* genome.

| **Type** | | **Copy** | **Average length (bp)** | **Total length (bp)** | **% of genome** |
| --- | --- | --- | --- | --- | --- |
| miRNA | | 80 | 124.39 | 9,951 | 0.000396 |
| tRNA | | 1,285 | 74.86 | 96,199 | 0.025358 |
| rRNA | rRNA | 2,275 | 280.17 | 637,379 | 0.025358 |
|  | 18S | 347 | 1121.48 | 389,154 | 0.015483 |
|  | 28S | 813 | 141.78 | 115,264 | 0.004586 |
|  | 5.8S | 203 | 155.8 | 31,628 | 0.001258 |
|  | 5S | 912 | 111.11 | 101,333 | 0.004032 |
| snRNA | snRNA | 882 | 111.06 | 97,955 | 0.003897 |
|  | CD-box | 652 | 105.77 | 68,960 | 0.002744 |
|  | HACA-box | 18 | 128 | 2,304 | 0.000092 |
|  | splicing | 212 | 125.9 | 26,691 | 0.001062 |

## Supplementary Table 10. Statistical results of repeat sequences.

| **Type** | **Repeat Size(bp)** | **% of genome** |
| --- | --- | --- |
| TRF | 60,980,312 | 4.459431 |
| RepeatMasker | 209,193,906 | 15.298148 |
| RepeatProteinMask | 314,744,165 | 23.016936 |
| De novo | 771,808,868 | 56.441634 |
| Total | 858,835,374 | 62.805798 |

## Supplementary Table 11. Statistics of repeat sequences in *D. chrysotoxum*.

|  | **RepBase TEs** | | **TE Proteins** | | **De novo** | | **Combined TEs** | |
| --- | --- | --- | --- | --- | --- | --- | --- | --- |
|  | **Length** | **%in** | **Length** | **%in** | **Length** | **%in** | **Length** | **%in** |
|  | **(bp)** | **Genome** | **(bp)** | **Genome** | **(bp)** | **Genome** | **(bp)** | **Genome** |
| DNA | 13,675,689 | 1.00009 | 16,523,144 | 1.208322 | 9,467,177 | 0.692325 | 28,778,046 | 2.104511 |
| LINE | 38,965,431 | 2.849504 | 79,065,271 | 5.781967 | 71,655,790 | 5.240118 | 132,460,276 | 9.686692 |
| SINE | 231,405 | 0.016922 | 0 | 0 | 0 | 0 | 231,405 | 0.016922 |
| LTR | 158,486,236 | 11.589945 | 219,653,630 | 16.063057 | 690,356,601 | 50.485109 | 726,773,563 | 53.148246 |
| Other | 5,284 | 0.000386 | 0 | 0 | 0 | 0 | 5,284 | 0.000386 |
| Unknown | 0 | 0 | 0 | 0 | 6,571,394 | 0.48056 | 6,571,394 | 0.48056 |
| Total | 209,193,906 | 15.298148 | 314,744,165 | 23.016936 | 765,020,013 | 55.945172 | 837,103,567 | 61.216572 |

*LINE, long interspersed nuclear element; SINE, short interspersed element; LTR, long terminal repeat.

*Denovo+Repbase denotes transposable elements identified by RepeatMasker (<http://www.repeatmasker.org>) with default options after RepeatModeler/RepeatScout/Piler/LTR_finder software used with RepBase database prediction.

*TE proteins were transposable elements identified in the genome through the annotation of Repeat ProteinMask software using the RepBase database.

*Combined TEs involved a combination of the above two methods.

*Unknown repeat sequences could not be clustered by Repeat Masker.

## Supplementary Table 12. Statistical results of functional annotation.

| **Values** | **Total** | **Nr** | **Swissprot** | **KEGG** | **KOG** | **TrEMBL** | **Interpro** | **GO** | **Overall** |
| --- | --- | --- | --- | --- | --- | --- | --- | --- | --- |
| Number | 30,044 | 26,809 | 19,186 | 18,667 | 17,414 | 27,269 | 22,736 | 5,462 | 27,576 |
| Percentage | - | 89.23% | 63.86% | 62.13% | 57.96% | 90.76% | 75.67% | 18.18% | 91.78% |

## Supplementary Table 13. Gene Ontology (GO) enrichment results of significantly expanded gene families in *D. chrysotoxum* (see separate files).

## Supplementary Table 14. Statistical results of whole gene collinearity analysis.

| **Species 1** | **Species 2** | **Genes** | **Collinear blocks** | **Mean genes in one block** | **Collinear genes** | **Percentage (%)** |
| --- | --- | --- | --- | --- | --- | --- |
| *D. chrysotoxum* | *D. chrysotoxum* | 30045 | 231 | 6.67 | 2800 | 9.32 |
| *D. chrysotoxum* | *D. catenatum* | 56836 | 1149 | 10.95 | 24550 | 43.19 |
| *D. chrysotoxum* | *P. equestris* | 56516 | 838 | 13.44 | 21881 | 38.72 |
| *D. chrysotoxum* | *P. aphrodite* | 49718 | 277 | 39.93 | 21592 | 21592 |

**Supplementary Table 15. List of MADS-box genes identified in *D. chrysotoxum*.**

| **Gene ID** | **Gene name** | **ORF (bp)** | **Protein (aa)** | **Subfamily** |
| --- | --- | --- | --- | --- |
| Maker100372 | Dch100372 | 1614 | 537 | A |
| Maker100375 | Dch100375 | 1302 | 433 | A |
| Maker100381 | Dch100381 | 375 | 124 | AGL6 |
| Maker100385 | Dch100385 | 951 | 316 | ANR1 |
| Maker102565 | Dch102565 | 711 | 236 | AP3 |
| Maker10265 | Dch10265 | 576 | 191 | AP3 |
| Maker10266 | Dch10266 | 909 | 302 | AP3 |
| Maker105383 | Dch105383 | 1050 | 349 | AP3 |
| Maker105478 | Dch105478 | 348 | 115 | Bs |
| Maker109055 | Dch109055 | 1047 | 349 | C/D |
| Maker109817 | Dch109817 | 1179 | 392 | C/D |
| Maker110150 | Dch110150 | 855 | 284 | C/D |
| Maker111025 | Dch111025 | 909 | 301 | C/D |
| Maker111658 | Dch111658 | 636 | 211 | E |
| Maker111667 | Dch111667 | 636 | 211 | E |
| Maker111954 | Dch111954 | 1056 | 351 | E |
| Maker112050 | Dch112050 | 723 | 240 | E |
| Maker28116 | Dch28116 | 315 | 104 | M alpha |
| Maker41263 | Dch41263 | 633 | 210 | M alpha |
| Maker53142 | Dch53142 | 759 | 252 | M alpha |
| Maker54373 | Dch54373 | 519 | 173 | M alpha |
| Maker56037 | Dch56037 | 657 | 218 | M alpha |
| Maker56241 | Dch56241 | 732 | 243 | M alpha |
| Maker58058 | Dch58058 | 672 | 223 | M alpha |
| Maker61232 | Dch61232 | 1050 | 349 | M alpha |
| Maker61954 | Dch61954 | 879 | 292 | M alpha |
| Maker63932 | Dch63932 | 753 | 250 | M alpha |
| Maker69282 | Dch69282 | 699 | 232 | M gamma |
| Maker72535 | Dch72535 | 597 | 198 | M gamma |
| Maker73171 | Dch73171 | 750 | 249 | M gamma |
| Maker74460 | Dch74460 | 753 | 270 | M gamma |
| Maker76017 | Dch76017 | 723 | 240 | M gamma |
| Maker81481 | Dch81481 | 567 | 188 | M gamma |
| Maker82541 | Dch82541 | 837 | 278 | M gamma |
| Maker83303 | Dch83303 | 870 | 189 | M gamma |
| Maker83866 | Dch83866 | 822 | 273 | M gamma |
| Maker87204 | Dch87204 | 684 | 227 | M gamma |
| Maker87678 | Dch87678 | 675 | 224 | M gamma |
| Maker87759 | Dch87759 | 1053 | 350 | M gamma |
| Maker89530 | Dch89530 | 579 | 192 | MIKc* |
| Maker95519 | Dch95519 | 735 | 244 | MIKc* |
| Maker96265 | Dch96265 | 972 | 323 | PI |
| Maker96746 | Dch96746 | 657 | 218 | PI |
| Maker97788 | Dch97788 | 483 | 160 | SOC1 |

## Supplementary Table 16. The expression of genes in carotenoid biosynthesis pathway and regulatory mechanisms.

| **Name** | **Gene ID** | **Bud** | **IniOp** | **Blm** | **Fd** | **Stem1** | **Stem2** | **Stem3** | **Leaf1** | **Leaf2** | **Leaf3** |
| --- | --- | --- | --- | --- | --- | --- | --- | --- | --- | --- | --- |
| PSY | Maker83370 | 53.39 | 57.00 | 20.46 | 14.59 | 50.10 | 59.93 | 39.89 | 109.36 | 153.96 | 118.06 |
| PSY | Maker32731 | 14.72 | 63.11 | 46.08 | 32.67 | 0.39 | 0.30 | 0.08 | 0.26 | 0.39 | 0.15 |
| OR | Maker66469 | 65.48 | 173.14 | 101.22 | 84.43 | 24.56 | 36.17 | 34.84 | 79.59 | 88.12 | 74.60 |
| OR-LIKE | Maker111682 | 58.11 | 94.25 | 87.47 | 89.43 | 82.64 | 112.95 | 96.44 | 112.61 | 125.38 | 131.89 |
| CLPB3 | Maker84981 | 36.50 | 41.75 | 31.87 | 26.92 | 142.88 | 85.05 | 111.53 | 54.14 | 51.20 | 85.45 |
| DXS | Maker86880 | 70.33 | 124.46 | 49.46 | 38.93 | 31.10 | 44.91 | 31.31 | 27.27 | 28.06 | 30.94 |
|  | Maker86884 | 67.91 | 114.46 | 34.95 | 35.59 | 26.00 | 34.35 | 32.14 | 28.36 | 25.62 | 29.98 |
| PDS | Maker54122 | 145.57 | 1414.56 | 352.87 | 413.04 | 11.51 | 17.22 | 15.57 | 34.49 | 33.17 | 25.29 |
| Z-ISO | Maker73577 | 42.91 | 134.42 | 50.56 | 46.09 | 14.31 | 20.00 | 18.92 | 28.31 | 25.56 | 22.71 |
| ZDS | Maker100941 | 128.70 | 745.29 | 474.05 | 410.99 | 41.46 | 47.12 | 46.07 | 80.63 | 76.92 | 80.38 |
| CTRISO | Maker59413 | 16.81 | 42.88 | 50.78 | 64.35 | 6.74 | 8.43 | 7.55 | 13.22 | 12.49 | 12.75 |
| LYCB | Maker67572 | 17.48 | 37.87 | 29.20 | 29.11 | 16.32 | 21.33 | 23.67 | 35.37 | 41.20 | 35.22 |
| LYCE | Maker70264 | 17.52 | 12.91 | 3.82 | 3.37 | 15.61 | 16.85 | 11.60 | 20.93 | 19.49 | 20.34 |
| PGM48 | Maker110151 | 15.71 | 66.34 | 45.55 | 55.19 | 18.49 | 18.18 | 19.05 | 80.31 | 69.09 | 66.28 |
| CYP97A3 | Maker86659 | 112.66 | 479.24 | 238.46 | 276.03 | 26.78 | 33.35 | 27.41 | 99.70 | 95.78 | 80.63 |
| CYP97C1 | Maker116351 | 21.30 | 28.70 | 12.98 | 9.30 | 4.64 | 6.41 | 5.64 | 20.10 | 16.03 | 17.19 |
| CYP97C1 | Maker51218 | 1.05 | 0.30 | 0.44 | 0.12 | 0.49 | 0.46 | 0.40 | 1.90 | 2.15 | 2.13 |
| CYP97C1 | Maker121734 | 24.85 | 37.86 | 13.68 | 14.77 | 2.80 | 3.34 | 3.53 | 14.31 | 11.79 | 12.41 |
| BCH | Maker65814 | 7.80 | 14.03 | 6.76 | 5.97 | 31.84 | 32.88 | 30.25 | 9.69 | 14.59 | 12.11 |
| BCH | Maker109431 | 157.50 | 1800.20 | 902.77 | 659.40 | 0.00 | 0.12 | 0.07 | 0.03 | 0.03 | 0.00 |
| ZEP | Maker82479 | 8.60 | 4.14 | 2.75 | 3.39 | 7.02 | 20.94 | 11.07 | 33.67 | 31.80 | 27.32 |
| VDE | Maker89122 | 23.16 | 24.49 | 20.53 | 17.69 | 8.67 | 8.81 | 9.88 | 19.88 | 23.92 | 14.98 |
| ABA4-NXS | Maker113481 | 78.34 | 37.51 | 20.26 | 20.42 | 5.72 | 4.58 | 6.79 | 8.53 | 8.32 | 6.72 |
| ABA4-NXS | Maker118392 | 16.81 | 9.29 | 0.72 | 2.13 | 0.12 | 0.51 | 0.28 | 0.07 | 0.00 | 0.00 |
| XAT | Maker66153 | 315.36 | 87.18 | 28.54 | 1.78 | 0.92 | 1.02 | 1.79 | 0.98 | 1.34 | 1.02 |
| CCD4 | Maker99416 | 0.00 | 0.00 | 0.00 | 0.00 | 0.00 | 0.00 | 0.00 | 0.00 | 0.00 | 0.00 |
|  | Maker107515 | 0.00 | 0.00 | 0.00 | 0.00 | 0.00 | 0.00 | 0.00 | 0.00 | 0.00 | 0.00 |
|  | Maker99401 | 0.03 | 0.00 | 0.00 | 0.03 | 0.87 | 2.04 | 0.95 | 3.82 | 1.03 | 2.14 |
|  | Maker114782 | 0.19 | 0.00 | 0.03 | 0.00 | 0.10 | 0.15 | 0.10 | 16.90 | 6.76 | 14.90 |
|  | Maker99378 | 0.00 | 0.00 | 0.00 | 0.00 | 0.00 | 0.00 | 0.00 | 0.29 | 0.00 | 0.00 |
|  | Maker99339 | 0.00 | 0.00 | 0.00 | 0.00 | 0.00 | 0.00 | 0.00 | 0.00 | 0.00 | 0.00 |
|  | Maker107530 | 0.00 | 0.00 | 0.00 | 0.00 | 0.00 | 0.00 | 0.00 | 0.00 | 0.00 | 0.00 |
| CCD8 | Maker114213 | 1.43 | 0.24 | 0.00 | 0.07 | 11.72 | 2.15 | 4.06 | 0.00 | 0.00 | 0.00 |
|  | Maker117913 | 1.37 | 0.12 | 0.00 | 0.00 | 2.88 | 0.66 | 1.01 | 0.03 | 0.00 | 0.03 |
| CCD7 | Maker116019 | 0.03 | 0.00 | 0.00 | 0.03 | 0.24 | 0.66 | 0.87 | 0.00 | 0.00 | 0.00 |
|  | Maker67149 | 0.00 | 0.00 | 0.00 | 0.00 | 0.00 | 0.00 | 0.00 | 0.00 | 0.00 | 0.00 |
|  | Maker67132 | 1.95 | 0.57 | 1.26 | 0.76 | 0.04 | 0.00 | 0.00 | 0.02 | 0.13 | 0.00 |

Green color: the gene expression increased gradually; orange colour: the gene expression decreased gradually.

## Supplementary Table 17. The expression of gene in abscisic acid biosynthesis pathway.

| **Name** | **Gene ID** | **Bud** | **IniOp** | **Blm** | **Fd** | **Stem1** | **Stem2** | **Stem3** | **Leaf1** | **Leaf2** | **Leaf3** |
| --- | --- | --- | --- | --- | --- | --- | --- | --- | --- | --- | --- |
| NCED | Maker56123 | 0.06 | 0.63 | 0.48 | 0.79 | 3.68 | 2.08 | 7.04 | 0.09 | 0.13 | 0.00 |
|  | Maker69003 | 11.70 | 6.27 | 5.73 | 11.88 | 2.92 | 2.51 | 4.98 | 4.64 | 5.79 | 4.66 |
| SDR1 | Maker95296 | 19.52 | 22.47 | 19.40 | 17.82 | 10.13 | 9.95 | 11.69 | 12.90 | 13.57 | 12.16 |
| SDR2 | Maker93730 | 0.55 | 1.07 | 1.25 | 1.26 | 0.00 | 0.10 | 0.00 | 85.85 | 56.40 | 26.52 |
| AAO | Maker111635 | 8.37 | 3.71 | 2.79 | 1.57 | 1.25 | 1.12 | 1.53 | 0.88 | 0.72 | 0.76 |
| MOCO | Maker60410 | 6.10 | 6.41 | 5.27 | 5.96 | 6.51 | 6.83 | 7.49 | 9.32 | 8.92 | 8.02 |
|  | Maker60384 | 4.64 | 6.59 | 3.24 | 5.29 | 3.58 | 3.02 | 3.68 | 5.64 | 5.62 | 5.18 |
|  | Maker59665 | 20.74 | 22.52 | 18.66 | 19.65 | 8.85 | 11.42 | 11.21 | 11.08 | 11.51 | 11.40 |
|  | Maker58183 | 10.12 | 6.58 | 3.14 | 3.56 | 1.23 | 0.53 | 0.70 | 5.75 | 8.77 | 3.22 |
|  | Maker13158 | 13.63 | 15.32 | 6.38 | 2.16 | 0.52 | 0.13 | 0.05 | 0.48 | 0.56 | 0.28 |

Green color: the gene expression increased gradually; orange color: the gene expression decreased gradually.

## Supplementary Table 18. The expression of genes in Ethylene biosynthesis pathway and regulatory mechanisms.

| **Name** | **Gene ID** | **Bud** | **IniOp** | **Blm** | **Fd** | **Stem1** | **Stem2** | **Stem3** | **Leaf1** | **Leaf2** | **Leaf3** |
| --- | --- | --- | --- | --- | --- | --- | --- | --- | --- | --- | --- |
| SAM1/2 | Maker79017 | 2101.39 | 2316.12 | 3275.54 | 2719.31 | 308.90 | 789.44 | 317.14 | 328.35 | 315.76 | 216.95 |
| ACS10/12 | Maker60889 | 11.32 | 19.27 | 12.26 | 11.43 | 6.95 | 8.72 | 8.00 | 19.22 | 17.67 | 16.68 |
|  | Maker120658 | 24.70 | 22.37 | 15.94 | 18.38 | 62.36 | 75.97 | 57.69 | 93.35 | 85.03 | 96.68 |
| ACS9/5/4/8/11 | Maker105598 | 0.12 | 0.41 | 0.08 | 0.29 | 0.03 | 0.11 | 0.03 | 0.09 | 0.03 | 0.00 |
| ACS2/1/6 | Maker109266 | 0.78 | 0.74 | 0.08 | 0.40 | 0.00 | 0.00 | 0.03 | 0.06 | 0.09 | 0.00 |
| ACS7 | Maker75695 | 0.87 | 3.19 | 1.91 | 1.19 | 0.00 | 0.00 | 0.10 | 0.03 | 0.03 | 0.17 |
|  | Maker97433 | 0.00 | 0.00 | 0.00 | 0.04 | 0.00 | 0.00 | 0.00 | 0.00 | 0.00 | 0.00 |
|  | Maker66290 | 0.05 | 0.16 | 0.81 | 20.30 | 0.03 | 0.00 | 0.00 | 0.00 | 0.00 | 0.04 |
| ACO4 | Maker29641 | 899.31 | 2456.81 | 975.47 | 1680.31 | 18.86 | 37.94 | 52.93 | 1124.47 | 1164.27 | 158.14 |
| ACO5 | Maker77098 | 0.49 | 0.15 | 0.36 | 0.07 | 0.00 | 0.00 | 0.00 | 0.16 | 0.00 | 0.05 |
|  | Maker77030 | 4.12 | 0.45 | 4.83 | 1.22 | 0.09 | 0.00 | 0.05 | 0.47 | 0.56 | 0.16 |
| EIN2 | Maker78483 | 28.22 | 36.07 | 39.67 | 43.12 | 45.37 | 40.31 | 48.17 | 35.33 | 41.69 | 38.08 |
| CTR1 | Maker00834 | 32.77 | 29.26 | 23.52 | 26.19 | 25.23 | 26.54 | 32.46 | 23.25 | 27.25 | 24.16 |

Green color: the gene expression increased gradually;
